# The Plant‐to‐Plant Circular Strategy: Coupling Photodegradation and Phytoremediation With Plant‐Based Nanomaterials for Plastic Degradation

**DOI:** 10.1002/advs.76789

**Published:** 2026-07-24

**Authors:** Haoran Liu, Lena Ciric, Yuheng Wang, Ziru Pei, Manpreet Bhatti

**Affiliations:** ^1^ UCL Department of Civil Environmental and Geomatic Engineering London WC1E 6BT UK; ^2^ Shaanxi Key Laboratory of Qinling Ecological Intelligent Monitoring and Protection Department of Ecology and Environment, School of Life Science and Technology Northwestern Polytechnical University Xi'an Shannxi 710129 People's Republic of China

**Keywords:** circular bio‐economy, photodegradation, phytoremediation, plant‐based nanomaterials, plastic, soil microorganisms

## Abstract

The environmental recalcitrance of widely used plastics, exemplified by polyethylene (PE), creates a core remediation trade‐off between efficient yet ecologically risky synthetic catalysts and benign but slow phytoremediation. To bridge this gap, plant‐based nanomaterials (PB‐NMs) as dual‐function catalysts are introduced for a sustainable “Plant‐to‐Plant” circular strategy with Alfalfa (*Medicago sativa* L.). Synthesized from biomass via a low‐energy process, PB‐NMs achieved 16.09% PE degradation in 28 days under UV‐A and 11.35% in 90 days within soil alongside alfalfa, which is the highest efficiency compared to two other commercial carbon NMs. Systematic variation of plastic film placement reveals preliminary evidence of concentration effects, spatial and temporal differences, and the dependence of NM catalytic activity on plants during degradation. Microbial and functional gene analyses further suggest that, depending on spatial and temporal conditions, PB‐NMs can activate distinct degradative bacterial communities and oxidative pathways. Collectively, our findings suggest a safe, closed‐loop system where PB‐NMs, UV light, and alfalfa synergistically degrade PE with no detectable trophic transfer under the evaluated conditions. This biocompatible synergy could potentially enable decentralized, household‐level plastic remediation, offering a conceptual pathway to transform a centralized burden into a scalable, eco‐positive practice for the circular bio‐economy.

## Graphical abstract

Plant‐based nanomaterials derived from alfalfa serve as dual‐function catalysts, coupling photodegradation and phytoremediation to degrade polyethylene under UV‐A light and in soil. The nanomaterials work synergistically with alfalfa and soil microbes, achieving efficient plastic breakdown without trophic transfer. This closed‐loop strategy offers a potential pathway for decentralized, household‐level plastic remediation.

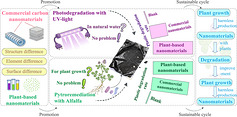

## 1 Introduction

In the global crisis of plastic pollution, polyethylene (PE) stands out as both a symbolic and quantitatively dominant pollutant [1, 2]. It represents approximately 60% of the global plastic waste stream [3]. Once released into the environment, PE consistently exhibits the highest detected concentrations in contaminated settings across regions worldwide [4]. Meanwhile, its pervasive accumulation disrupts ecological stability and human health [5, 6], a consequence driven primarily by its biotoxicity [7, 8]. However, conventional physico‐chemical remediation methods for PE are often energy‐intensive, costly, and risk causing secondary pollution [9, 10]. Meanwhile, the policies and management of plastic pollution on a global scale are also chaotic and unsustainable [3, 4]. Thus, this situation demands innovative remediation strategies that are aligned with ecological recovery and designed for accessibility and inclusive societal engagement.

Phytoremediation is a low‐intervention strategy aligned with natural processes [11]. Plants can stabilize or degrade pollutants through root activity, exudates, and associated microbial communities [12, 13]. However, when confronted with highly inert synthetic polymers like PE, the innate plant‐microbe system often exhibits exceedingly slow and inefficient degradation [13]. Recently, nanotechnology has offered new avenues for plastic degradation [14, 15]; for instance, photocatalytic nanomaterials (NMs) can generate reactive oxygen species under light to cleave polymers [16, 17]. However, most engineered NMs, including carbon and metal NMs, suffer from poor environmental biocompatibility, aggregation tendencies, and potential unknown toxicological effects on soil biota and plants [15, 18, 19]. This has restricted the application of NMs, particularly in agricultural [20] or household settings, creating a fundamental dilemma between their high catalytic efficiency and ecological safety, which severely hinders their practical use in ecosystem restoration [21]. In contrast, our prior study has demonstrated that plant‐based NMs (PB‐NMs) offer a sustainable and green alternative, characterized by negligible toxicity, potential for localized production, and promising applicability in the eco‐remediation of certain organic pollutants (such as dyes) and heavy metals [such as mercury (Hg)] [19, 22]. Meanwhile, plant‐derived biomass materials at non‐NM scales have been shown to efficiently capture plastics via physical adsorption [23]. However, PB‐NMs have not been reported for photodegradation or bioremediation of persistent synthetic polymers like PE. Therefore, the central question arises: whether and how these eco‐compatible PB‐NMs can be harnessed to effectively bridge nanotechnology with remediation, overcoming the toxicity barrier that has plagued conventional approaches.

To bridge this gap, this study aims to construct a near‐harmless remediation process for PE, advancing a sustainable materials strategy grounded in renewable feedstocks and ecological compatibility. We directly convert plant biomass into PB‐NMs without resorting to harsh chemicals, thereby adhering to the principles of benign‐by‐design chemistry and minimal environmental burden [24]. These PB‐NMs are deployed back into phytoremediation systems, establishing a “from‐plants, for‐plants” cycle. This cycle is designed to utilize PB‐NMs to achieve catalytic effects while maintaining environmental safety, directly enhancing indigenous microorganisms within the system. Driven by a dual‐pathway synergy of photodegradation and phytoremediation, this closed‐loop system is expected to overcome the biocompatibility bottleneck associated with conventional engineered NMs. This integration represents a shift from conventional phytoremediation by coupling photocatalytic activation with microbial assimilation, forming a closed‐loop system where PB‐NMs act not as passive additives but as active mediators of oxidative and biological transformation. This systems‐level approach distinguishes the work from prior studies focusing solely on either material catalysis or plant uptake.

## 2 Results and Discussion

### 2.1 Synthesis and Characterization of PB‐NMs

**FIGURE 1:**
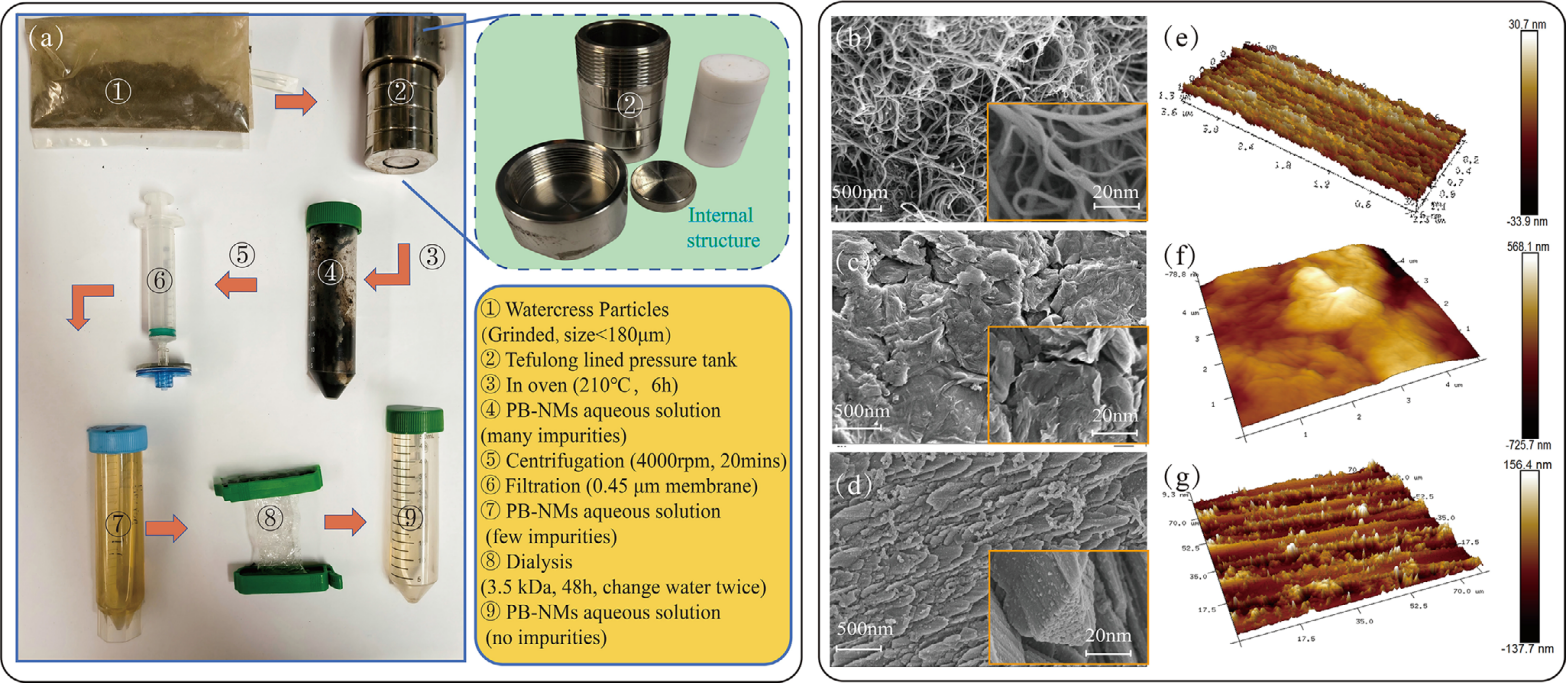
Preparation, characterization of PB‐NMs, and comparison with common mass‐produced NMs. (a) Preparation process and reaction conditions; (b‐d) SEM for (b) CNTs, (c) FGO and (d) PB‐NMs; (e‐g) AFM for (e) CNTs, (f) FGO, (g) PB‐NMs.

Utilizing alfalfa (*Medicago sativa* L.) as the sole precursor, PB‐NMs were synthesized via a green and facile hydrothermal pyrolysis process. As illustrated in Figure 1a, the entire laboratory‐scale procedure could be efficiently completed within 72 h through 9 defined steps, demonstrating the practicality and accessibility of this synthesis route [18, 25, 26]. The Zeta potential of the three types of NMs was as follows: −25.6 ± 2.1 mV (CNTs), −32.8 ± 1.9 mV (FGO), and −40.4 ± 3.3 mV (PB‐NMs). This indicates that PB‐NMs have better colloidal stability. This is consistent with the observation that PB‐NMs in Milli‐Q (MQ) water presented a yellow, non‐precipitating, semi‐transparent mixed liquid state. It is also consistent with the observation that soluble PB‐NMs > FGO > CNTs. This characteristic makes it easier to freely migrate in natural aquatic or moist soil environments, offering a distinct advantage for applications in water and soil pollution remediation.

As shown in Table S1, the specific surface areas of CNTs, FGO, and PB‐NMs are 89.77, 63.60, and 846.33 m^2^ g^−1^, respectively, analyzed by Brunauer‐Emmett‐Teller (BET). Figure 1b,c confirmed that both Multi‐walled carbon nanotubes (CNTs) and Few‐layer graphene oxide (FGO) exhibited their expected structures by scanning electron microscope (SEM). In Figure 1d, PB‐NMs exhibit a lamellar structure similar to FGO on a relatively macroscopic scale (500 nm) and a unique triangular prism structure with a diameter of <20 nm on a relatively microscopic scale (20 nm). These morphological characteristics are influenced by the synthesis conditions and the type of plant precursor used [19].

Atomic force microscopy (AFM) further revealed distinct surface structures among the three NMs. CNTs showed a high density of triangular pyramidal protrusions, while FGO exhibited a smooth, featureless surface over a significantly larger scanning area, aligning with findings from our earlier study [27]. PB‐NMs demonstrated a unique surface topography, characterized by numerous needle‐like protrusions observable at a scale slightly larger than that of CNTs. The formation of these needle‐like structures is likely attributable to the incomplete carbonization and directed assembly of biomacromolecules, such as cellulose and lignin, present in the plant‐based precursor under hydrothermal conditions at elevated temperature and pressure [28, 29].

**FIGURE 2:**
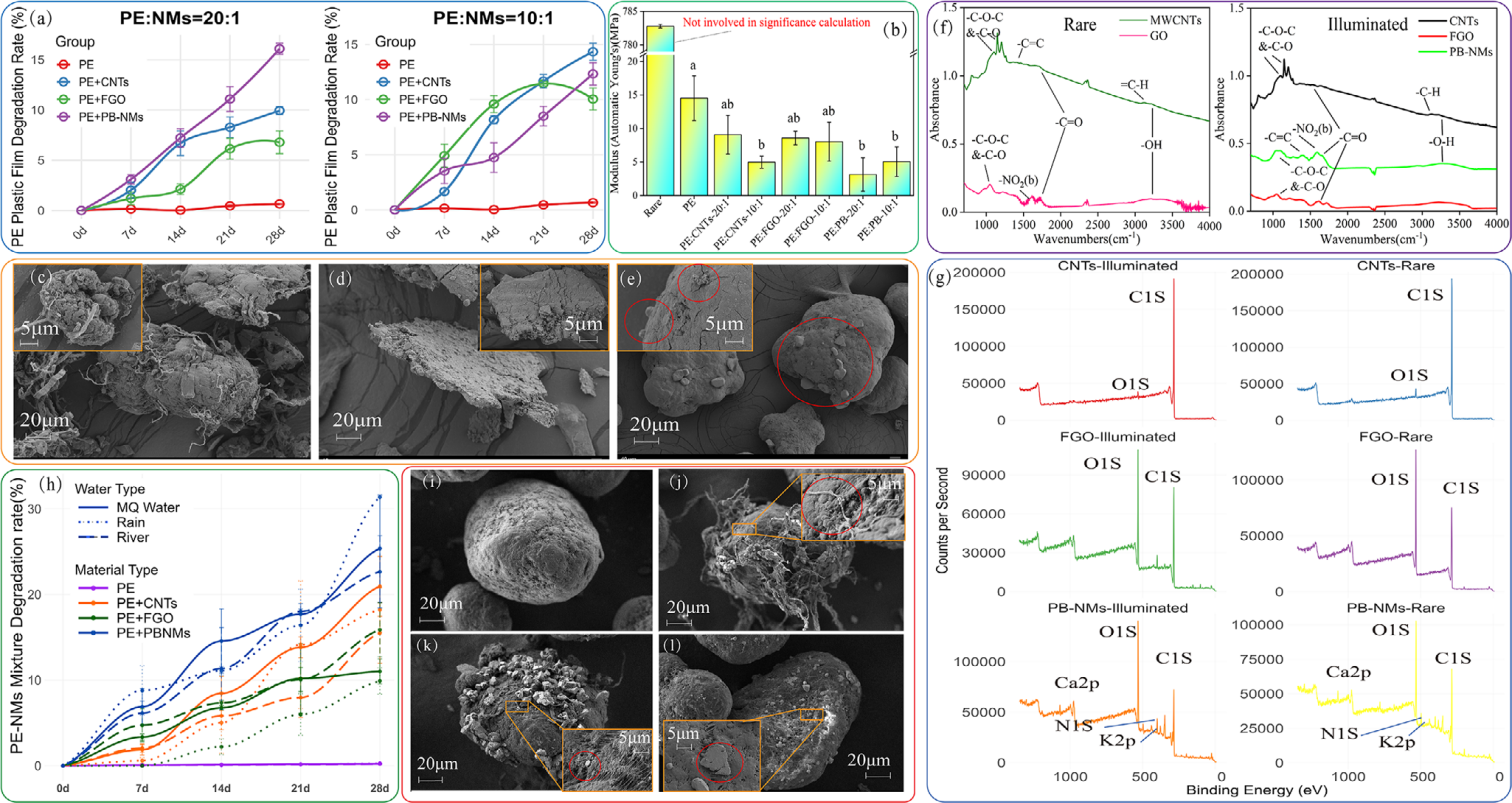
Effects and principles of plastic photodegradation catalyzed by NMs and UV‐LLS. (a) 28‐day dynamic degradation curves of PE film (b) The strength of the PE film at 28 days (“Rare” = control group); (c‐e) SEM of NMs on the PE film after 28d photodegradation, including (c) CNTs, (d)FGO and (e)PB‐NMs; (f) FT‐IR of NMs before and after 28d photodegradation; (g) XPS of NMs before and after 28d photodegradation; (h) 28‐day dynamic degradation curve of PE MPs and NMs mixture in water; (i‐l) SEM of PE MPs after 28d photodegradation with NMs, including (i) PE only, (j) PE+CNTs, (k) PE+FGO, and (l) PE+PB‐NMs.

Unlike CNTs and FGO, which exhibited only basic oxygen‐containing groups as shown in Figure 2d and Supplementary Information I (SI‐I) Section 1, PB‐NMs showed a broader and more intense suite of functional signatures. These signatures were mostly distinct NO_2_ vibrations, reflecting a higher degree of surface functionalization that may underpin their enhanced catalytic behavior [30]. This trend is corroborated by the X‐ray photoelectron spectroscopy (XPS) presented in Figure 2e, where PB‐NMs display detectable signals corresponding to nitrogen (N) 1s, potassium (K) 2p, and calcium (Ca) 2p, in addition to the dominant C1s and oxygen (O) 1s peaks. The emergence of these heteroatomic signals can be attributed to the inherent composition of the plant precursors, which introduce N‐containing moieties, as well as mineral elements such as potassium and calcium, into the carbon framework during the carbonization process [31, 32]. These elements are absent in pristine carbon NMs like CNTs and FGO, further highlighting the distinct chemical features imparted by the biomass template. Nevertheless, carbon and oxygen remain the principal constituents across all three NMs, underscoring the central role of the carbon backbone and oxygenated functional groups in defining their chemical identities.

Beyond their structural and chemical uniqueness, PB‐NMs benefit from a green, low‐cost, and operationally simple synthesis route. Compared with other biomass‐derived NMs, such as carboxylated cellulose nanofibers (C‐CNFs) requiring harsh oxidation [33] or biomass‐derived 3D graphene foams (3DGFs) produced via high‐temperature pyrolysis [20], PB‐NMs avoid hazardous reagents and show reduced biotoxicity, offering a more sustainable alternative for catalytic applications. Collectively, these results demonstrate that the hierarchical morphology, enriched surface functionalities, and heteroatom incorporation intrinsic to PB‐NMs act synergistically to endow the material with enhanced catalytic activity.

### 2.2 Photocatalytic Degradation of PE by PB‐NMs

UV‐LLS (UV LED light strip) irradiation experiments were designed to evaluate the degradation of PE, as shown in Figure S15a,b. Figure 2a shows the dynamic results of double‐layer black PE films under direct exposure.

After 28 days of photodegradation, all NM‐catalyzed groups exhibited significantly higher degradation than the control (PE‐only), which showed only 0.65 ± 0.11% at 28 days. In contrast, under visible light and dark irradiation, all groups showed less than 0.2% degradation, as shown in the SI‐II Raw data table. This difference arises because the saturated C═C and C─H backbones of polyethylene possess higher bond energies, requiring higher‐energy UV photons to initiate scission [10, 16].

These results confirm that both UV and NMs are essential elements for the efficient degradation of PE films [18, 34, 35], and demonstrate that UV‐LLS, as a generally harmless UV type, is effective for initiating the breakdown of PE films. As identified in many previous reports, the degradation products were ethylene and propionic acid under NMs‐catalyzed photodegradation [1, 10]. Regarding NM type and dosage, the group with a PE:NMs mass ratio of 20:1 showed that PB‐NMs achieved the highest degradation rate of 16.09 ± 1.09% after 28 days, outperforming CNTs (9.94 ± 0.80%) and FGO (6.79 ± 2.27%). Moreover, the PB‐NMs group displayed a nearly linear and sustained degradation throughout the 0–28 day period, suggesting continued catalytic potential, while the degradation curves of CNTs and FGO tended to level off. When the dosage was increased (PE:NMs = 10:1), the degradation rate of PB‐NMs after 28 days was slightly lower than that of CNTs by approximately 2%, but still higher than that of FGO. These observations collectively highlight that PB‐NMs retain robust photocatalytic activity even at higher loading levels, remaining comparable to CNTs and superior to FGO.

The mechanical strength of the film is an important indicator of its susceptibility to degradation [21, 36]. In Figure 2b and Figure S15c, tensile testing based on Young's modulus showed that after 28 days, all UV‐exposed films experienced a marked loss of mechanical integrity, even the sample without added NMs declined from an initial value of 782.79 ± 0.26 MPa (Rare) to 14.53 ± 3.33 MPa (PE) after UV exposure, demonstrating the strong weakening effect of UV on PE film strength. Then, PE films treated with NMs exhibited additional decreases in tensile strength compared with PE‐only. Especially, low‐dose CNTs and both PB‐NMs dose groups showed significant differences compared to the PE‐only. The similarity between strength‐loss patterns and degradation patterns further supports the role of PB‐NMs in catalyzing PE fragmentation. Figure S1c indicates that the efficiency of plastic photodegradation will significantly decrease and become unstable under natural environments, such as soil cover or dust influence [37].

After 28 days, the NMs on the PE film surface were analyzed by BET, Fourier Transform Infrared (FT‐IR), SEM, and XPS to further investigate the mechanisms through which NMs promote photodegradation. Figure 2c–e directly shows the NMs and the PE particles on the 28‐day PE film surface. The identification of PE particles was based on comparisons with the industrialized PE particles shown in Figure 2i. CNTs are observed adsorbed onto PE particles, consistent with the common adsorption of NMs onto plastic [38]. Compared with their original shapes as shown in Figure 1b, treated CNTs formed noodle‐like aggregates, while these plastic particles were different in both shape and size. For FGO, only aggregated FGO was detected, with no clear evidence of PE‐like particles, which is consistent with the substantial decrease in surface area after the reaction shown in Table S1. For PB‐NMs, no significant aggregation was observed. Instead, the PB‐NMs were well‐dispersed across the surface of the plastic particles, which is consistent with the fact that the biomass components have a high adsorption capacity for plastic [23]. These observations suggest that at least CNTs and PB‐NMs enhance the photodegradation of PE films by promoting the formation of more MPs.

Figure 2f compares the surface functional groups of the NMs before and after the reaction. PE chains contain a few unsaturated (C═C) bonds, like vinyl groups. These sites are easily oxidized by radicals, forming unstable hydroperoxides that convert into stable, UV‐absorbing carbonyl groups to enable UV degradation [21, 39]. NMs may accelerate this process, as their surface functional groups can catalyze radical production or act as radicals directly [31, 40]. For CNTs and FGO, the peaks generally appeared at their original positions and retained similar shapes, whereas PB‐NMs showed significant changes, including an overall decrease in peak intensity. This suggests that some of the PB‐NMs surface functional groups, such as –NO_2_(a), –C–O–C–, and –C–H, may be consumed during the photodegradation process, likely through electron transfer or bond cleavage reactions [27, 30, 41]. In contrast, the surface functional groups of CNTs and FGO either did not actively participate in the process or maintained higher catalytic stability [26, 34]. FT‐IR analysis of the PE films before and after photodegradation, as shown in Figure S1a, revealed that the pristine Rare PE film exhibited the lowest baseline and the weakest characteristic peaks. After 28 days of UV exposure, all NM‐loaded films showed elevated baselines and increased absorbance in the 3000–3600 cm^−1^ region [hydroxyl/hydroperoxide (‐OH/‐OOH) groups], indicating surface photo‐oxidation [26].

Figure 2g shows the XPS comparison of NMs before and after the reaction. For CNTs and FGO, the carbon content (both peak intensity and area) remained nearly unchanged, while the oxygen content decreased. This may indicate that some oxygen‐containing groups, such as –OH and –C–O–C–, were involved in the reaction, potentially contributing to polymer chain scission and oxidation [16, 41]. For PB‐NMs, both C and O content increased, while N and K content decreased. This may reflect the simultaneous catalytic action and strong adsorption of degraded PE fragments by PB‐NMs, consistent with BET in Table S1. The contribution of N and K to electron regulation and active‐site formation further explains the superior catalytic performance of PB‐NMs [40, 42]. As shown in Figure 1b, XPS quantification further compared the surface elemental composition of the PE films before and after degradation. Relative to the pristine Rare PE (O1s/C1s = 0.166), the photoaged PE without NMs showed a slight increase (O1s/C1s = 0.192), confirming slow abiotic oxidation. Among NM‐loaded groups, PB‐NMs induced the highest surface oxygen content (O1s/C1s = 0.378), followed by CNTs (0.149) and FGO (0.161). Weak N1s and S2p signals were preserved only in the PB‐NMs group, suggesting residual PB‐NMs on the PE surface that may contribute to its catalytic activity. These XPS results are consistent with the FT‐IR observations, jointly demonstrating that PB‐NMs promote the most effective surface oxidation [10, 31], in agreement with the gravimetric degradation rates as shown in Figure 2b.

Building on the morphological evidence of NM‐PE interactions (Figure 2c–e), Figure 2h shows 28‐day degradation dynamics in different water types. The degradation efficiencies showed minimal dependence on water type, demonstrating that this catalytic system is resilient across natural aqueous environments. Within each water type, PB‐NMs consistently achieved significantly higher degradation rates (p < 0.05) than CNTs and FGO. The enhancement was most notable in rainwater (31.37 ± 5.98%) and remained substantial in river water (22.66 ± 4.22%). These results show that PB‐NMs maintain strong catalytic performance in natural water systems, extending their utility beyond controlled laboratory conditions.

Figure 2i–l present micrographs of NMs and MPs after reaction. The control group showed no significant changes after 28 days, consistent with negligible degradation. All three NM‐treated groups exhibited distinct surface cracking, consistent with catalytic stress concentration and oxidative chain scission [21, 36]. Figure 2j–l show CNTs, FGO, and PB‐NMs interacting with MPs. PB‐NMs showed a dispersion behavior similar to Figure 2e, again supporting their effective catalytic engagement. Dark controls in Figure S2b–d showed NM agglomeration, indicating that UV and NMs act synergistically [14, 43]. Thermogravimetric analysis (TGA) in Figure S2 shows that PB‐NMs‐catalyzed degradation did not produce more stable refractory residues, consistent with morphological observations [20, 33, 44]. This finding aligns with the morphological characteristics of PE particles observed in Figure 2c,e, supporting the role of PB‐NMs in enhancing degradation efficiency without promoting undesirable stabilization pathways.

In summary, CNTs and PB‐NMs capture suspected degradation intermediates, but PB‐NMs additionally utilize their –OH and C‐based surface groups to actively drive oxidative fragmentation. Through this synergistic action, PE films are progressively transformed into microplastic fragments. These fragments subsequently degrade into gases such as ethylene, liquid products including propionic acid [1], and finally mineralize into CO_2_, H_2_O, and small organic acids [16, 21]. Therefore, the combination of PB‐NMs and UV‐LLS enables a simplified and environmentally compatible degradation pathway, which may be scalable to household or small‐system applications. This decentralization of remediation technology could reduce the carbon footprint associated with plastic waste transport and centralized processing, thereby aligning localized treatment with broader ecological impact mitigation [19].

Collectively, these results show that PB‐NMs not only accelerate photodegradation but do so through mechanisms that are inherently compatible with natural systems. Their dispersion, surface chemistry, and heteroatom‐driven reactivity enable efficient polymer fragmentation with no detectable additional environmental risks to alfalfa under the tested conditions. This establishes PB‐NMs as a sustainable catalytic alternative capable of supporting decentralized, low‐energy plastic remediation.

### 2.3 Phytoremediation of Soil PE Films by Alfalfa and PB‐NMs

**FIGURE 3:**
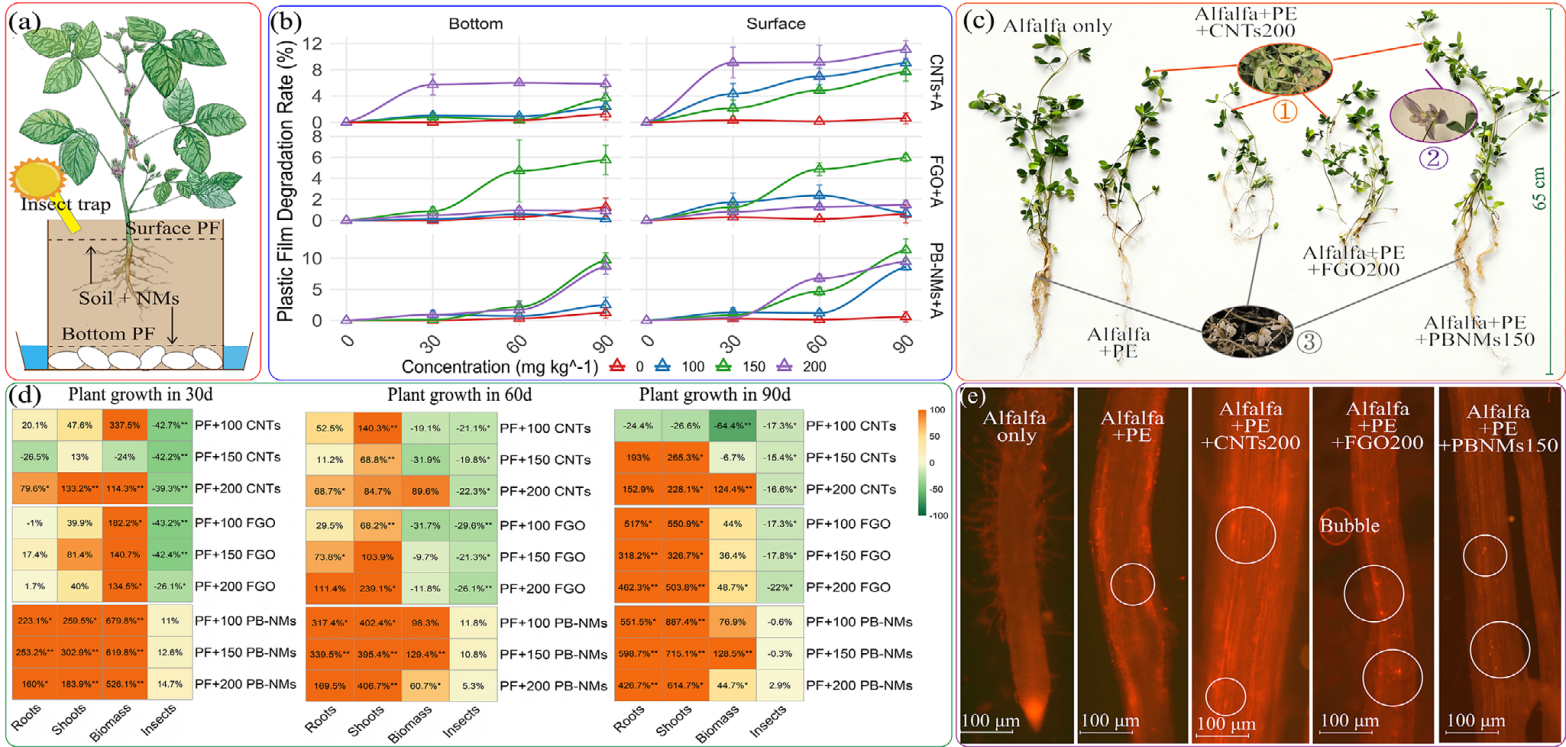
PE phytoremediation based on three types of NMs and alfalfa, including (a) Schematic diagram of the device and important growth characteristics of alfalfa; (b) Dynamic degradation curves of surface and bottom PE film from 0 to 90 days at three dosages of three types of NMs; (c) Growth comparison of the plants with the highest degradation rate group and Alfalfa only among the three NMs types; (d) Plant growth indicators based on heat map responses; (e) Plant roots stained with fluorescence using Nile red and the circles represent the abnormal luminous positions compared with Alfalfa only. The number combined with the NMs represents the concentration. For example, CNTs200 represents CNTs 200 mg kg^−1^. Note: In Figure 3c, ① shows yellow leaves in all PE‐added groups; ② shows flowers only in the PB‐NMs 150 mg kg^−1^ at 90d; ③ shows Root nodules in the Alfalfa only, CNTs and PB‐NMs groups at 60 and 90d.

Based on the 90‐day degradation experiment of PE films in alfalfa+soil systems with three types of NMs at three concentrations, the results are shown in Figure 3. While Figure S3 shows that none of the NMs alone caused significant toxicity to germination, the addition of PE films significantly decreased germination, and PB‐NMs uniquely mitigated this inhibition, significantly alleviating the toxic effects of PE.

Figure 3a presents a schematic cross‐section of the experimental setup. Then in Figure 3c, the actual images show flowering and nodule development in alfalfa, both of which are generally considered important indicators of healthy plant growth [45]. Flowering was observed only in the 150 mg kg^−1^ PB‐NMs treatment. Pink block‐shaped nodules were detected only in the 60 and 90‐day samples of CNTs and PB‐NMs. All PE‐added groups showed yellowing and drying of leaves, affecting plants of all heights. From 0 to 90 days, the soil pH remained at 7.0 ± 0.5 for all groups.

Figure 3d highlights the differences in plant growth indicators between NM‐treated groups and the Control. At 30 days, PB‐NMs at all three concentrations already showed significant increases in plant height, root length, and biomass compared with the Control, while conventional NMs showed limited promotion, with only CNTs200 showing comparable effects. By 90 days, the PB‐NMs groups demonstrated dramatic increases in root length (598.7%, *p* < 0.001), plant height (887.4%, *p* < 0.001), and biomass (128.5%, *p* < 0.01). In the FGO group, significant improvements were also observed in most indicators except for biomass. In contrast, plant growth in the CNTs groups, especially CNTs100, was lower than in the Control, as plants were generally smaller, though CNTs200 showed a significant advantage. Despite this, CNTs and FGO consistently maintained a significant reduction in soil fly populations. All trapped insects were identified as fungus gnats (*Bradysia *spp., Sciaridae). This suggests that these two traditional NMs may possess potential biotoxicity, inhibiting plant growth while simultaneously suppressing the survival of soil insect larvae [46, 47]. Then, Figure 3e shows the results of the Nile red fluorescence staining of the root system. Abnormal fluorescent color blocks compared with Alfalfa only were observed in all PE‐added groups, which have been reported as indicative of PE presence [8, 13]. The application of NMs, especially PB‐NMs and FGO, enhanced PE uptake into alfalfa roots, although its presence in nodules, stems, and leaves (Figure S5) could not be conclusively confirmed due to autofluorescence. Future studies employing pre‐staining of PE could further validate these observations [48].

Consistent with the patterns observed in plant growth, the dynamic degradation data shown in Figure 3b demonstrate that all NM‐treated groups exhibited significantly higher degradation rates than the Control from 0 to 90 days, both at the surface and at the bottom layers. At the soil surface, CNTs200 showed an early and pronounced effect, reaching 9.09 ± 2.33% degradation at 30 days, followed by only a slight increase to 11.08 ± 1.34% by 90 days. In contrast, other treatments displayed continuous increases over time, highlighting the importance of plant growth in driving plastic degradation. Among them, FGO achieved a maximum degradation rate of less than 6%, while PB‐NMs reached 11.35 ± 1.76% (150 mg kg^−1^) and 9.47 ± 0.44% (200 mg kg^−1^). The superior and sustained growth associated with PB‐NMs suggests a synergistic effect between enhanced plant growth and PB‐NMs‐mediated degradation.

At the bottom layer, during actual plant growth at 60d, roots gradually extended through the surface film and intertwined with it, as illustrated in Figure S4d. As a result, during the 60–90 d period, the bottom‐layer films came under direct influence of plant roots. Correspondingly, increased degradation rates were recorded in FGO150 and all PB‐NMs groups, with PB‐NMs 150 and 200 reaching 9.71 ± 1.15% and 8.71 ± 1.27%, respectively, slightly exceeding their own surface degradation rates. In contrast, CNTs showed little root‐related impact. For instance, CNTs200 already achieved 5.75 ± 1.57% degradation in 30 days but exhibited no substantial changes between 30 and 90 days. Figure S6a presents the tensile strength of PE films, with significant differences between surface and bottom layers observed in the CNTs200, PB‐NMs150, and PB‐NMs200 90d. This indicates that although the overall degradation rates were similar, the degree of aging of the PE films was more pronounced at the surface [49, 50]. These results collectively suggest that plant growth enhances PE aging and degradation [51, 52], while this effect is most strongly enhanced by PB‐NMs. However, CNTs and FGO do not follow this trend, possibly because it acts alone, resulting in a lower degradation rate.

Figure S6b,c further revealed that PB‐NMs+Plants induced the strongest surface oxygen enrichment and unique N/S signals on PE films by FT‐IR and XPS, indicating active involvement of PB‐NMs in rhizoremediation. In contrast, CNTs+Plants showed only moderate oxidation without heteroatom fingerprints. These chemical modifications, absent without plants, confirm the long‐term synergy between PB‐NMs and the alfalfa rhizosphere. Furthermore, the high colloidal stability of PB‐NMs (zeta potential = ‐40.4 mV) enables their migration through the soil matrix, facilitating degradation beyond immediate contact zones and supporting the environmental feasibility of this system under real‐world conditions.

### 2.4 Analysis of Microbial Communities in NM‐Driven Phytoremediation

**FIGURE 4:**
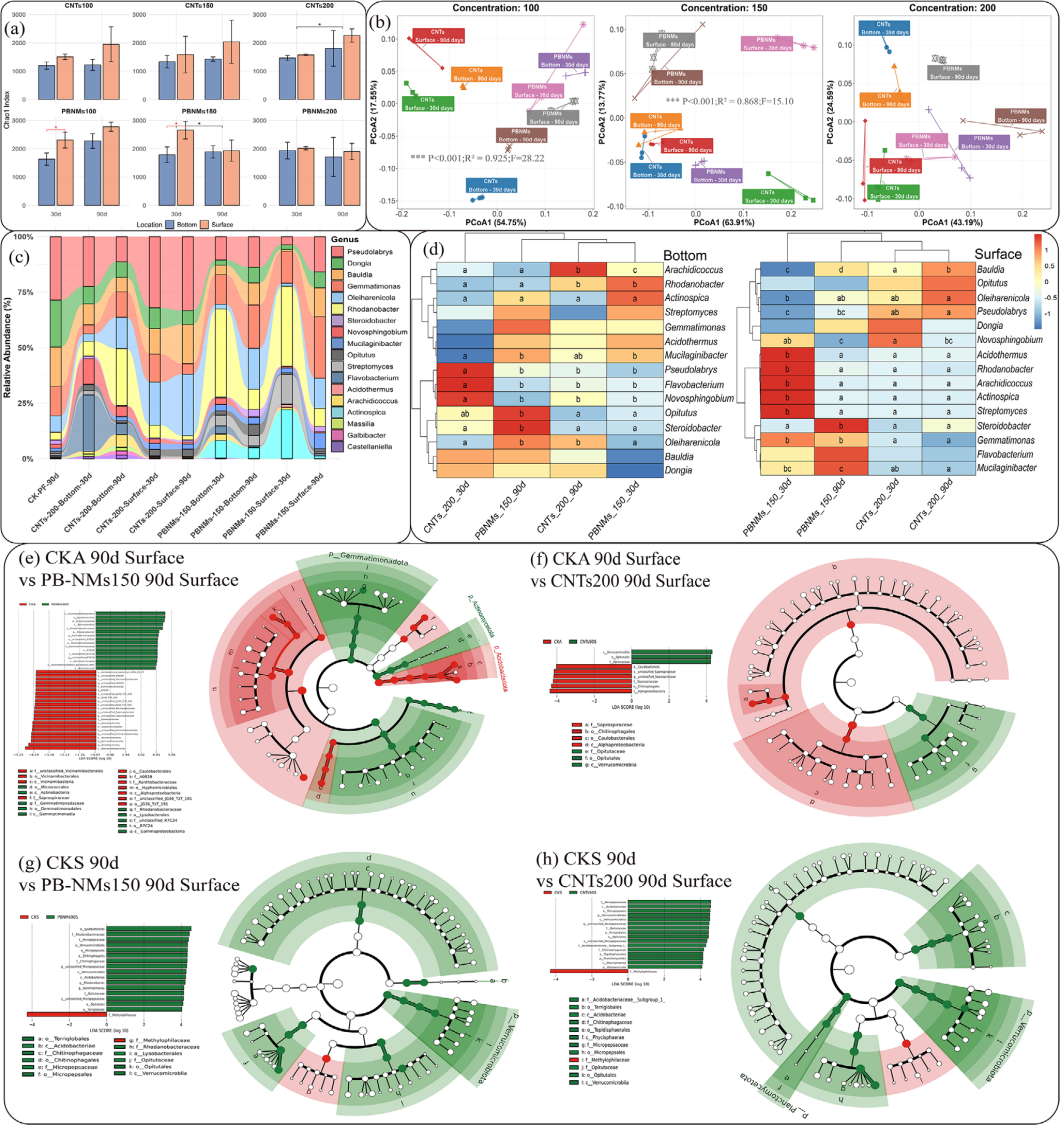
Comparison of microbial communities before and after phytoremediation, including (a) α diversity at dynamic scale in the two highest degradation rate groups with Chao1 as a representative indicator. (b) β diversity at concentration and dynamic scale with PCoA as the representative indicator; (c) Relative abundance alluvial diagram of two highest degradation rate groups at the Genus level; (d) Relative abundance heatmaps and significance of two highest degradation rate groups at the Surface and Bottom scales and Genus levels; (e–h) Phylogenetic tree based on LEfse, in the (e,f) two highest degradable groups vs CKA (alfalfa only + PE) control group and (g,h) two highest degradable group vs CKS (PE only) control group.

**FIGURE 5:**
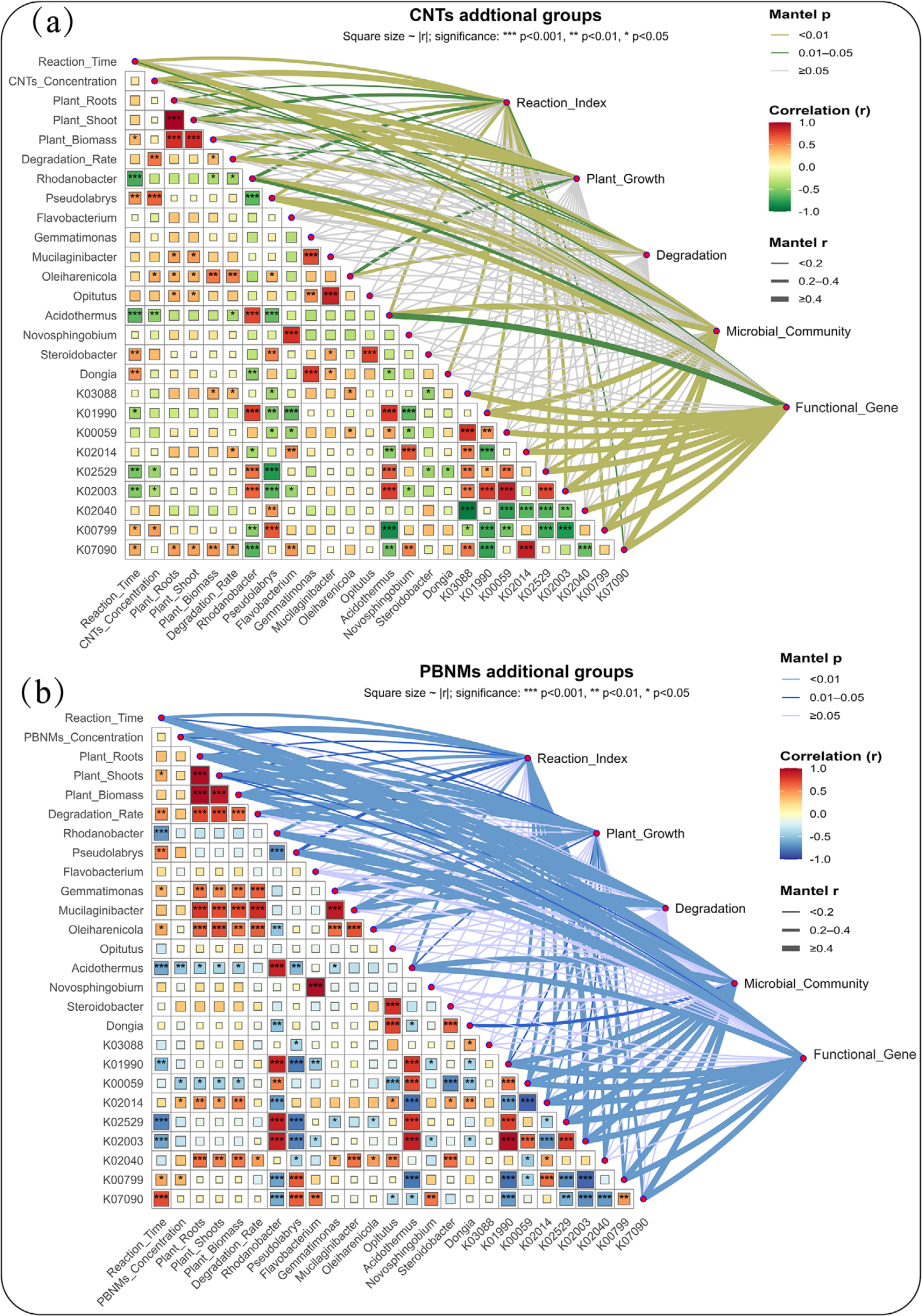
Correlation heatmap and Mantel analysis of reaction time, NMs concentration, PE degradation rate, potential degrading bacteria, and significantly changed KEGG, including (a) CNTs additional group; (b) PB‐NMs additional group. In the lower left section of the analysis is the correlation heatmap, while the upper right section displays the Mantel test results, which consist of five blocks. Reaction time and concentration were grouped into the Reaction Index; three plant indicators were grouped into Plant Growth; degradation rate was grouped into Degradation; all potential degrading bacteria were grouped into Microbial Community, and all significantly changed genes were grouped into Functional Gene. Taking (a) Plant Growth as an example, the lines indicate significant correlations (based on the Mantel test p‐value) between this block and each indicator. For instance, it shows a significant correlation with *Oleiharenicola* (p = 0.01–0.05, represented by a dark green line).

The high‐throughput sequencing results of microbial communities are presented in Figures 4, 5, with particular attention given to CNTs and PB‐NMs improved high‐degrading groups based on Figure 3b. Figure 4a illustrates the differences in α‐diversity under three concentration levels of CNTs and PB‐NMs, using Chao1 as the representative index [53]. The other five commonly used diversity indices exhibited similar trends and are provided in Figure S7a–e. According to the Chao1 results, microbial communities in the surface samples were consistently more abundant than those in the bottom samples under all PB‐NMs concentrations. This difference was most pronounced in PB‐NMs150, which showed significant temporal and spatial effects (p < 0.05), indicating that PB‐NMs at this concentration substantially promoted microbial community growth. A similar trend was observed in the CNTs treatments. These results correspond well with the observed degradation rate patterns and further confirm the positive influence of plants on microbial community development under plastic stress [50, 52].

Figure 4b presents the β‐diversity differences among the three concentration levels, analyzed using Principal Coordinate Analysis (PCoA). The Non‐metric Multidimensional Scaling (NMDS) results showed similar patterns and are provided in Figure S7f. At all concentrations, CNTs consistently showed separate clustering of bottom and surface samples, regardless of incubation time (30 or 90 days). In contrast, PB‐NMs displayed a different trend. At 100 and 200 mg kg^−1^, surface and bottom samples remained similar at both time points, whereas at 150 mg kg^−1^, the 30‐day surface and bottom samples were clearly separated but became closer again at 90 days. These results indicate that in the CNTs treatments, plant growth influenced the surface microbial communities but had limited impact on the bottom [54], which corresponds with the slower plant growth observed in the CNTs group shown in Figure 3. However, in the PB‐NMs treatments, enhanced plant growth appeared to regulate the microbial communities in the PB‐NMs150 group, leading to greater convergence at 90 days. This suggests that the degradation in the PB‐NMs group was jointly driven by PB‐NMs and Alfalfa, whereas in the CNTs group, degradation was primarily driven by CNTs with a secondary contribution from Alfalfa [19, 45], which is supported by Figure S7g.

Figure 4c directly shows the relative abundance differences at the genus level between the highest‐degradation groups (CNTs200 and PB‐NMs150) and the control group. Relative abundances for all experimental and control groups across four taxonomic ranks are provided in Figure S8a–d. In the CNTs treatment, compared to the control group, there were marked increases in *Pseudolabrys*, *Oleiharenicola*, *Mucilaginibacter*, and *Opitutus*. With respect to the control, the PB‐NMs group showed significantly elevated abundances of *Gemmatimonas*, *Rhodanobacter*, and *Actinospica*, most of which have potential plastic degradation functions, as shown in Table 1. Figure S8 demonstrates that CNTs and PB‐NMs can regulate different microorganisms and that the concentration of NMs also leads to significant changes in the community. Regarding the principle of biodegradation, microbes can employ enzymes to degrade plastic into monomers, which are transported into the cell by specific monomer‐binding transporters and ultimately fed into the Tricarboxylic Acid Cycle (TCA) [9].

**TABLE 1:** Listed Genus‐level microorganisms and degradation functions or sensitivity to plastics.

Name	Target/Sources of coercion	Shape/status	Media/Machine	Materials or Plants	Function/Reason	References
Plastic degradation‐direct correlation						
*Rhodanobacter*	PE	Film	Soil	—	Plastic degradation	[55]
*Gemmatimonas*	HDPE, PLA and PHBV^1^	Pellet	Freshwater	—	Plastic degradation	[56]
*Novosphingobium*	PS	NPs^1^	Wastewater	—	Plastic resistance and degradation	[57]
	PS	Foam	Epilittoral zone	Mangroves	Plastic degradation	[58]
Plastic degradation‐Indirect correlation						
*Pseudolabrys*	PBAT^1^	Film	Soil	—	The soluble sugar and nitrate concentration of plants	[59]
*Opitutus*	PE and PBAT	Film	Soil	Maize	N cycling increased	[60]
Degradation of other organic pollutants						
*Oleiharenicola*	Methane	Gas	Methane‐Fed Bioreactor	—	Methane‐derived carbon cycling	[61]
*Steroidobacter*	BaP^1^	—	Soil	Rice straw amendment	Degradation	[62]
	PCBs^1^	—	E‐waste‐contaminated soil	—	Degradation	[63]
*Flavobacterium*	BaP	—	Epilittoral zone	Mangroves	Degradation	[64]
*Mucilaginibacter*	Phenol	—	Wastewater	SWCNTs^1^	Degradation by EPS^1^	[65]
	Cellulose	—	Agar plates	—	Degradation by EPS	[66]
Reduced by plastic (Plastic sensitivity)						
*Gemmatimonas*	HDPE, PS and PP^1^	MPs	Soil	—	C/N reduced	[67]
*Acidothermus*	PE	MPs	Soil	—	C/N reduced	[68]
*Dongia*	Al‐coated PET^1^	Film	Soil	—	C reduced	[69]

*Note*: 1. PBAT is Poly (butylene adipate‐co‐terephthalate); HDPE is High‐Density Polyethylene; PLA is Polylactic Acid; PHBV is Poly(3‐hydroxybutyrate‐co‐3‐hydroxyvalerate); NPs is nanoparticles; BaP is Benzo[a]pyrene; PCBs is Polychlorinated Biphenyls; PP is Polypropylene; PET is Polyethylene Terephthalate; SWCNTs is Single‐Walled Carbon Nanotubes; EPS is extra cellular polymeric substances. 2. Microorganisms not listed in the table were not reported under the three conditions.

Figure 4d presents a heatmap comparing the differential microbial community compositions between the CNTs200 and PB‐NMs150 treatments. Cluster analysis revealed that the bottom samples exhibited irregular grouping patterns, indicating that the two NMs exerted distinct influences on the bottom microbial communities [69, 70]. In contrast, the surface samples showed clear clustering according to NM type, with PB‐NMs and CNTs forming separate branches, suggesting that plant‐driven effects contributed to the difference in microbial communities in the soil. Within the PB‐NMs 90‐day surface group, *Steroidobacter* and *Mucilaginibacter* demonstrated significantly higher relative abundances compared with both CNTs and PB‐NMs 30‐day samples. In the CNTs 90‐day surface group, the dominant genera were *Oleiharenicola*, *Bauldia*, and *Pseudolabrys*. Meanwhile, more than five microbial genera showed significant enrichment exclusively in the PB‐NMs 30‐day surface group, implying a transient but distinct community response to PB‐NMs exposure during the early stage of incubation. These patterns further highlight the different effects of CNTs and PB‐NMs on soil microbial dynamics, with PB‐NMs exhibiting a stronger temporal modulation of surface communities and a closer association with plant‐driven microbial selection [55, 71], which is also supported by Figure S9.

In general, the enrichment of these genera in high‐degradation treatments suggests that both CNTs and PB‐NMs, together with the associated plant growth, may selectively favor microbial taxa with enhanced metabolic capacities for organic pollutant transformation or polymer fragment assimilation [2]. This process appears to involve both the stimulation of plastic‐degrading microorganisms, such as *Rhodanobacter* [55, 72], and the protection or stabilization of plastic‐sensitive taxa, including *Gemmatimonas* [67], *Acidothermus* [68], and *Dongia* [69], whose survival might otherwise be compromised by plastic pollution. This interpretation is consistent with XPS data as shown in Figure S6c, where PB‐NMs+Plants produced the highest surface oxygen and N/S enrichment, linking NM surface chemistry to enhanced microbial activity.

Based on the observed interactions between plants and NMs, Linear discriminant analysis Effect Size (LEfSe) analysis was employed to further investigate how NMs alone and plant‐NM systems modulated the soil microbial communities [27, 69]. Figure 4e,f illustrates the significant microbial shifts induced solely by NM treatments. In PB‐NMs, *c_Gemmatimonadia* and its corresponding taxa at the family and order levels [49, 56], as well as *f_Rhodanobacteraceae* [55], were significantly increased compared to CKA. In CNTs, *f_Opitutaceae* and its corresponding species at the order level were significantly increased [60]. This is consistent with the genus‐level observations in Figure 4, demonstrating that these three degrading bacteria are significantly promoted by PB‐NMs and CNTs, respectively. This also suggests that the communities promoted by PB‐NMs and CNTs differ, potentially through different mechanisms. Figure 4g,h highlights the pronounced regulatory effects of the combined NM‐plant systems. The significant increase in the number of microorganisms demonstrated the advantages of the combined effects of plant‐NMs in maintaining the prosperity of the microbial community [7, 57, 64].

Additionally, Figure S10 shows that differences between CNTs and PB‐NMs, as well as between the bottom and surface samples, suggest that both the plants and the NMs primarily regulate the relative abundance of similar microbial taxa rather than directly altering the overall community structure [73]. Figure S11 uses the Venn diagram to visually demonstrate the differences in the effects of CNTs and PB‐NMs on the community, the improvement in plant growth in the PB‐NMs additional group, and the convergence of treatment groups at 90 days [74, 75], supporting the conclusions of Figures 4 and Figures S6–S9. Figure S12, using the ternary diagram, further demonstrates that the degradation‐promoting effects of CNTs and PB‐NMs are transient and persistent, respectively [27, 76], supporting Figure 4 and Figures S6 and S11.

The functional gene differences between the high‐degradation and control treatments, as well as their temporal variations, are illustrated in Figure S13. Table S4 summarizes the specific functions of Kyoto Encyclopedia of Genes and Genomes (KEGG) pathways significantly enriched in high‐degradation treatments and suggests a coordinated mechanism for plastic biodegradation. First, K00059 cleaves the aliphatic backbone via β‐oxidation, a process potentially initiated by iron‐dependent oxidases supported by K02003 [49, 77]. Then, the resulting hydrophobic intermediates are likely detoxified by K00799, while K07090 may liberate metabolic products to complete assimilation [9].

Building upon these findings, the correlation analysis in Figure 5 further revealed that, in the CNTs group, three degradative genera and two functional genes (including K00799) showed significant differences in their predicted relative abundance over time [55]. With increasing NM concentration, *Pseudolabrys* exhibited a further significant rise. Meanwhile, plant growth showed strong correlations with several microbial taxa that were independent of CNTs, a pattern also supported by the Mantel test.

In contrast, PB‐NMs promoted alfalfa growth and effectively alleviated PE‐induced phytotoxicity, thereby reshaping the soil microbiome in a plant‐dependent manner. PB‐NM concentration displayed a similar positive correlation with K00799, which is likely because this Glutathione S‐Transferase (GST) enzyme detoxifies harmful oxidation byproducts generated during plastic breakdown, a process that would intensify with higher NM concentration [49]. Both plant growth and the PE degradation rate (the highest among all treatments in the PB‐NMs groups) showed significant correlations (p < 0.01) with three potential degradative genera, all of which also showed significant associations with the predicted relative abundance of K02040. This suggests that PB‐NMs restructure microbial communities by enriching degradative taxa, with a predicted enrichment of transporters (K02040) and degradation‐related detoxification genes (K00799) as inferred from 16S rRNA data [45]. The Mantel test further showed that the reaction index and plant growth were significantly correlated with most KEGGs (p < 0.05).

Integrating these results with Figures 3, 4 and Figure S6b,c suggests that PB‐NMs act in synergy with alfalfa to enhance PE degradation. To be specific, PB‐NMs promote plant growth, and together they improve soil microbial community structure, thereby stimulating the proliferation of degradative microorganisms, including but not limited to *Rhodanobacter* and *Gemmatimonas*, and a predicted increase in the relative abundance of functional genes (such as K00799and K02040) to achieve a more environmentally friendly degradation process [78]. In contrast, CNTs and plants possibly act independently in facilitating degradation, with CNTs even exhibiting potential phytotoxic effects at higher concentrations. Hence, a “plant‐to‐plant” cycle based on alfalfa is strongly suggested.

### 2.5 Plant‐Powered Closed‐Loop and Outlook for PE Bioremediation

**FIGURE 6:**
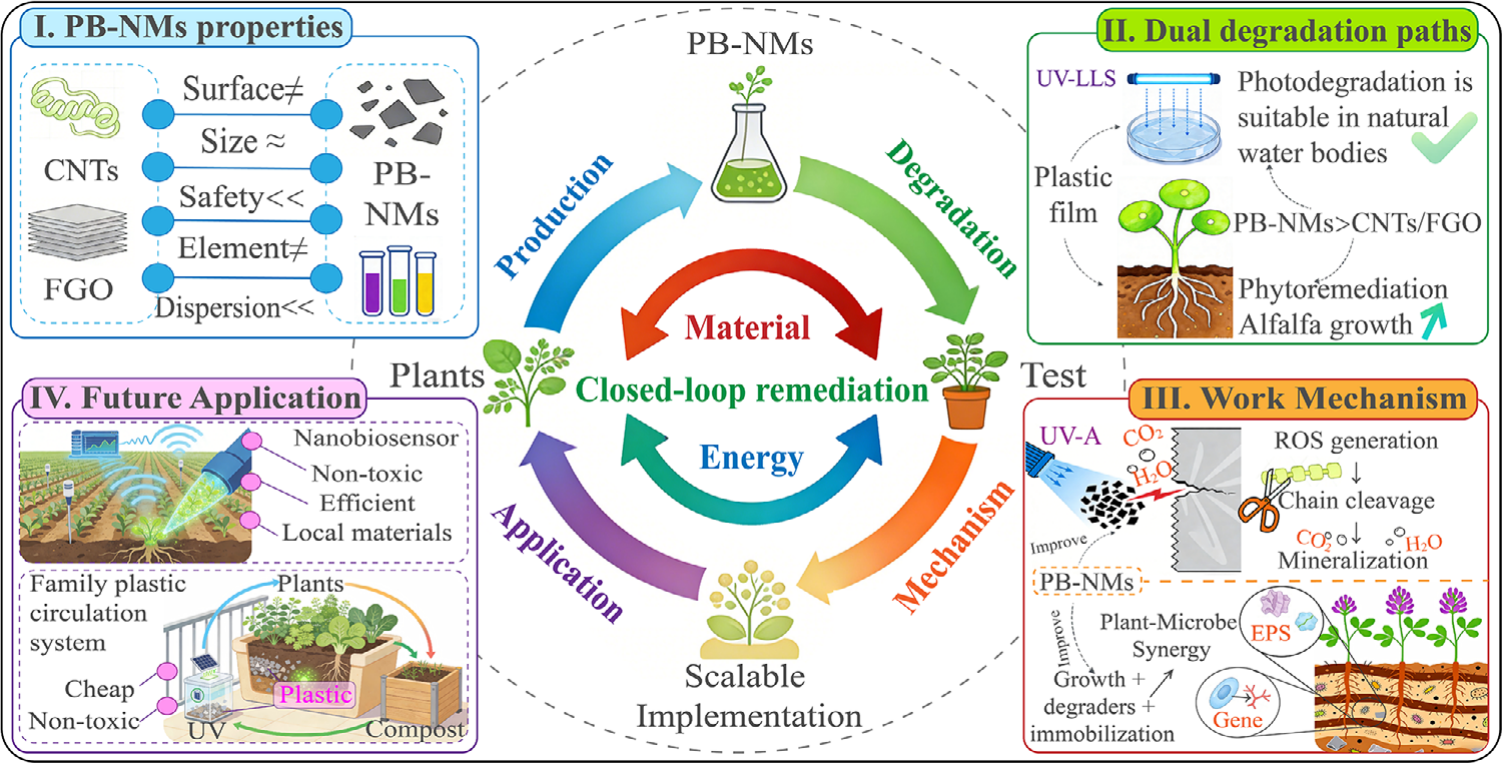
The Plant‐to‐Plant Closed‐Loop Remediation Cycle.

Based on the dual‐pathway experimental results and the consistent concept of harmlessness, this study ultimately proposes a transformative, closed‐loop framework for plastic phytoremediation, as illustrated in Figure 6. The “Plant‐to‐Plant Circular Strategy” uses PB‐NMs derived from alfalfa to drive dual‐pathway PE degradation while promoting plant growth and soil restoration. Importantly, data in Figure 3 and Figure S5 confirm that PE does not migrate to above‐ground tissues, indicating minimal risk of trophic transfer. This framework moves beyond the traditional linear “extract‐use‐dispose” model by establishing a self‐reinforcing ecological cycle, whose core innovation and safety assurance form the basis of a new remediation paradigm [11].

The sustainability and broad applicability of this plant‐centric cycle are among its most compelling advantages. The strategy demonstrates inherent material circularity: originating from plants, the non‐toxic (under the tested conditions) bio‐based PB‐NMs can safely remain in or return to the soil system after use, completing the loop. This eliminates the persistent environmental risk associated with conventional, non‐degradable engineered NMs like CNTs or FGO [18, 19]. This also avoids the energy‐intensive and costly process required in traditional NMs application methods, such as biological component extraction [1] and modified NMs production [18]. This inherent harmlessness, biocompatibility, and low consumption grant the system strong potential for applications like novel nano‐biosensors [24] and smart agriculture [42].

Most innovatively, design principles of this system enable a practical pathway toward decentralized, household‐level remediation. As the foundation, the low toxicity and efficacy of PB‐NMs make them suitable for small‐scale use (Figures 3, 4, 5, 6). Therefore, the entire process chain, from using household plant waste as potential PB‐NM feedstock in 72 h (Figure 1), to employing small‐scale solar UV units for plastic pretreatment or synchronous degradation (Figure 2), and finally to implementing enhanced phytoremediation in garden plots (Figure 3), can be adapted to the community or household level.

This system also demonstrates its positive effects in terms of reducing carbon emissions and achieving economic benefits. This decentralized implementation can reduce the carbon footprint of waste transport and empower individuals with a tangible tool for source‐level mitigation, transforming remediation from a centralized industrial process into a participatory, eco‐positive practice [79]. In addition, the sustainable design of this system could be further enhanced by integrating additional recycling steps, such as composting [45], and the utilization of degradation intermediates, such as ethylene [1], as potential energy sources.

In summary, this vision positions the work within a larger socio‐ecological context, where individual action gains new importance. It presents a future where safe, sustainable biological cycles allow individuals to mitigate local plastic pollution, contributing to ecosystem recovery. As our previous research [4] and related international policy research [3] indicate, individual efforts are becoming increasingly important in the face of global policy challenges. Therefore, our work establishes PB‐NMs as the cornerstone for a scalable, safe, and socially engaging circular bio‐economy, rather than merely a green material.

Looking ahead, this study points to two key strategic directions for future development. The successful remediation of PE strongly suggests that similar plant‐based cycles could be even more effective for other organic pollutants [34, 41]. Meanwhile, continued efforts to optimize material sources are essential for cost reduction and large‐scale practicality [19]. The limitations and outlook for field validation, photodegradation and bioremediation mechanisms, and PB‐NM future characterisation are discussed in SI‐I Section 4.

## 3 Conclusion

This study establishes a plant‐to‐plant circular strategy that shifts remediation from centralized, high‐intensity processes toward a distributed, ecological model. We synthesized PB‐NMs with a porous lamellar structure, which achieved a PE photodegradation efficiency of 16.09% under UV light, outperforming commercial benchmarks due to reduced aggregation and active surface groups. Critically, in soil, PB‐NMs at 150 mg kg^−1^ enhanced alfalfa biomass by 128.5% and promoted PE degradation (11.35% in 90 days) by broadly stimulating the soil microbiome and then forming a self‐reinforcing ecological loop. By seamlessly integrating photocatalysis with phytoremediation, PB‐NMs offer a dual‐function, low‐impact alternative to conventional NMs, demonstrating a viable pathway for household‐level, sustainable management of plastic pollution. In the future, the plant‐to‐plant circular system validated here for PE sets a foundational paradigm that could be extended to tackle a wider spectrum of anthropogenic pollutants, transforming distributed ecological remediation from a conceptual vision into a practical reality.

## 4 Experimental Section

All experiments in this research, except for characterization of NMs and High‐throughput sequencing, were conducted in the Environmental Engineering Laboratory, Department of Civil, Environmental and Geomatic Engineering, University College London, UK. All experiments were set up with three parallel samples. The original data are provided in the SI‐II Raw data table.

### 4.1 Synthesis and Characterization of PB‐NMs

PB‐NMs were synthesized from alfalfa. Alfalfa seeds were bought from Amazon and grown in a greenhouse from January 2023 to May 2023. Uniform aerial parts of plants were washed with deionized water, dried at 80°C for 8 h (wrapped in Al foil to exclude airborne dust), and then roasted in an oven at 100°C for 1 h until crispy by visual and olfactory inspections. The dried biomass was ground and sieved (100‐mesh, <150 µm). For each batch, 0.8 ± 0.1 g of the resulting powder was mixed with 10 ± 1.0 mL of MQ water in a Teflon‐lined autoclave (filling volume ca. 66.66% of the 15 mL vessel) and hydrothermally treated in a Teflon‐lined autoclave at 210°C for 6 h [26, 80]. After cooling, the product was centrifuged (4000 rpm, 20 min), filtered (0.45 µm), and dialyzed (3.5 kDa MWCO, 48 h with one water change) as shown in Figure 1a. The final PB‐NMs suspension was stored at 4°C with pH = 7.0 ± 0.5. After drying an aliquot of the suspension at 50°C to constant weight, the mean dry PB‐NM mass was 0.168 ± 0.018 g, corresponding to a yield of 21.0 ± 2.3% (based on 0.8 g starting biomass). After each batch of PB‐NMs is prepared, their weights are calculated individually rather than using this average value. This proportion is lower than that of the chemical synthesis method, but it is within the common range of the traditional hydrothermal synthesis method [81]. CNTs with an outer diameter of 5–20 nm and length of 10 µm and FGO with a thickness of 0.8–2 nm and lateral size of 5–10 µm (Brand: Shilpent) were used as control NMs. Both NMs have been reported to facilitate catalytic degradation processes [18, 27].

To comprehensively characterize the physicochemical properties of the as‐synthesized PB‐NMs, a suite of advanced analytical techniques was employed. Zeta potential was measured using a Brookhaven 90Plus PALS (Nanobrook) nanoparticle size and zeta potential analyzer (at pH 7, MQ water). The surface morphology was examined using a Zeiss SEM. The specific surface area and pore size distribution were determined via BET analysis using a Micromeritics ASAP 2460 surface area and porosity analyzer. The elemental composition and chemical states were probed by XPS on a Thermo Fisher Scientific ESCALAB Xi+ spectrometer. Surface functional groups were identified using FT‐IR on a Bruker VERTEX 80v spectrometer. The 3D topographic features were resolved through AFM using a Bruker Dimension Icon system in tapping mode.

### 4.2 Photodegradation Experiment of PE Film and MPs

A double‐layer black, commercial‐grade PE film for packaging was purchased from Amazon (Brand: Ramofy). The thickness is 20 µm, and the density range is 0.91–0.93 g cm^−3^. Laboratory‐grade PE microplastic particles (200 mesh) were obtained from Alibaba (Brand: Narry New Materials).

The gardening soil used for covering experiments was acquired from the B&Q supermarket (Brand: Verve Bulb Planting Compost). This standard peat‐free multipurpose compost consisting of composted green waste, wood fibre, and sand was employed. The characteristics included pH = 6.75 ± 0.75, organic matter = 67.5 ± 17.5%, total nitrogen (N), phosphorus (P), and potassium (K) ≥ 2%, respectively.

River water and rainwater samples were collected from the Lea Valley, London (51.59°N, 0.051°W) on January 27, 2025, which is the London water source area. No PE plastic contamination was detected above the detection limit in these water samples. Sampling points and investigation details can be found in SI‐I Section 3 and Figure S14.

After filtration, the water was stored at 4°C and used within 48 h. A UV‐LLS (Brand: Greenic) with a wavelength range of 380–400 nm (UV‐A level) and a simulated sunlight indoor LED tube (Brand: LOFTer, used in its 4500K daylight mode) were both purchased from Amazon. All lamps are located 50 cm above experimental samples, as shown in Figure S15a,b. The irradiance (E) of UV‐LLS is 190.99 µW·cm^−2^ based on the beam angle (120°). Under these conditions, an exposure of approximately 52 s at 50 cm corresponds to a dose of 10 mJ·cm^−2^. E of white light is 84.88 µW·cm^−2^ based on beam angle (120°), and about 118 s at 50 cm gives 10 mJ·cm^−2^. Photodegradation experiments were conducted in the light‐blocking device with a temperature detection device. The study included three exposure scenarios: (1) direct exposure of PE film, (2) soil‐covered exposure of PE film, and (3) aqueous exposure of PE microplastics. All experimental setups were performed in Petri dishes with three replicates per group and maintained for 28 days. Destructive sampling was carried out at three intervals: 7, 14, and 28 days. Both UV‐LLS and simulated sunlight lamps were used as light sources, with dark conditions serving as the control group. There was no significant change in temperature (25°C) throughout the entire experiment.

The first experiment was NM‐enhanced photodegradation of PE film. The weight ratios of PE film to NMs were set at a high concentration (10:1) and a low concentration (20:1). Because no prior studies were found in literature databases, including Google Scholar and Web of Science, as of July 11, 2025 (when this experiment concluded), the concentration of NMs was determined through pre‐experiments only, specifically selecting the level that achieved over 2% degradation within 48 h and 4% degradation within 7d. The mass of PE film used was 0.1 ± 0.01 g, corresponding to a square area of approximately 8 cm × 8 cm. The amounts of solid NMs added were 0.01 ± 0.001 g and 0.005±0.001 g, respectively. Throughout the experiment, the film surface was moistened with water to improve contact between plastic and NMs. This was done by applying 10 mL of MQ water at each 48 h interval using a calibrated pipette for each sample. The second experiment focused on NM‐promoted UV degradation of soil‐covered PE film. Under consistent experimental conditions, the PE film was covered with 0.1 g of soil to simulate photodegradation in natural environments. The third experiment entailed NM‐facilitated photodegradation of PE microplastics in an aqueous environment. A total of 0.1 ± 0.01 g of PE microplastic particles was introduced into 10 mL of three aqueous media, including MQ water, river water, and rainwater. The solid NM dosage was maintained at 0.01 ± 0.001 g. The samples were continuously mixed using an orbital shaker at 25°C with 300 rpm throughout the exposure period. The recovery rate of NMs and PE mixture is 100% ± 0.01%.

After the photodegradation, the treated PE films were sequentially rinsed with copious MQ water, then dried in a fume hood for 48 h and weighed. This rinse‐dry‐weigh cycle was repeated until consecutive measurements showed no significant mass change (defined as < 0.1 mg), indicating complete removal of surface residues. Then, films were weighed using an analytical balance to determine mass loss and calculate the degradation rate [82].

Subsequently, tensile tests were performed using a tensile tester based on the BS EN ISO 527‐1:2019 standard to characterize material strength as shown in Figure S15c. The rinsed NMs were air‐dried and subjected to FT‐IR, SEM, and AFM, while the recovered PE films were analyzed by FT‐IR and XPS to examine structural/morphological and surface chemical changes before and after degradation.

For the microplastic degradation process, the reacted mixtures were air‐dried, and the resulting solid residues were analyzed by TGA on a Setaram Themys One thermal analyzer to determine compositional changes and quantify microplastic degradation rates. The analyzer was heated to 800°C at a rate of 10°C/min.

### 4.3 Phytoremediation Experiment of PE Film

The phytoremediation experiment was conducted in a greenhouse equipped with plant growth lights, with temperature maintained at 25 ± 1°C. The nutrient soil and seeds used in this experiment were consistent with those in Sections 2.1–2.2. Alfalfa was selected due to its well‐documented efficacy in organic pollutant remediation [45]. A 7‐day germination test and a 45‐day hydroponic experiment with NMs addition were first conducted to identify suitable plant type and NM concentrations, as shown in Figures S3 and S4.

The soil‐cultivation setup is illustrated in Figure 3a. Each pot was filled with 700 ± 10 g of gardening soil mixed with three concentrations (100, 150, and 200 mg kg^−1^) of three NMs (CNTs, FGO, and PB‐NMs), underlain by a gravel layer at the bottom [76, 77, 82]. Two pieces of PE film (0.7 ± 0.01 g each, approximately 20 cm × 20 cm) were buried 2 cm above the gravel and 2 cm below the soil surface, respectively, ensuring full coverage of the pot cross‐section. Alfalfa was sown on the surface, and insect traps were installed. After germination, the number of plants was maintained at 25–30 per pot. For each treatment combination, three independent pots were prepared as biological replicates. Each pot served as a single experimental unit and was destructively sampled at each time point. No subsamples from the same pot were treated as independent replicates. Destructive sampling was conducted at 30, 60, and 90 days. Control groups included: (a) PE‐added groups without plants and without NMs; (b) PE‐added groups with plants but without NMs; (c) PE‐added groups without plants but with NMs. A blank group with plants but without PE or NMs was also established. Soil pH was monitored throughout the experiment, and moisture content was consistently regulated. During destructive sampling, surface soil, bottom soil, PE film, plant tissues, and insect traps were collected. Among them, soil samples were frozen at −80°C for subsequent DNA extraction. Others were treated immediately.

The degradation rate and characterization of the PE film were determined using the same method as in the photodegradation experiment. Plant samples were immediately analyzed for growth indicators including plant height, root length, and fresh biomass. After removing outliers (plants >10 cm taller than the pot average), we measured the longest individual with a ruler to two decimal places for plant height and root length. For plant biomass (wet weight), all plants from a pot were rinsed with MQ water until no soil particles were visibly adherent. After surface moisture was removed with absorbent paper, the biomass was weighed to an accuracy of 0.01 g using an analytical balance. Insects on traps were counted promptly. For each treatment, the values from three independent pots were then averaged to obtain the mean growth indicators as shown in SI‐II.

Furthermore, the plants designated for root length and shoot height measurements were dissected into root, shoot, leaf, and root nodule segments. These segments were then stained with Nile red dye (Brand: Bioisco) in the dark for 10 min [13], and observed and photographed using a Zeiss Imager.M2 fluorescence microscope equipped with an Axiocam 712 color camera.

### 4.4 High‐Throughput Sequencing of Soil Microorganisms in Phytoremediation Experiments

DNA was extracted from 0.5 g soil samples using the FastDNA Spin Kit for Soil (MP Biomedicals LLC, Ohio, USA), following the manufacturer's protocol. DNA concentration and purity were measured with a Nanodrop ND‐1000 UV–vis Spectrophotometer (NanoDrop Technologies, Wilmington, DE). The V4–V5 hypervariable region of the bacterial 16S rRNA gene was amplified with the primers 515F (5’‐GTGCCAGCMGCCGCGG‐3’) and 907R (5’‐CCGTCAATTCMTTTRAGTTT‐3’) [27]. PCR amplification, library preparation, and sequencing were conducted by Novogene, Cambridge, UK. Sequencing libraries were constructed using the NEBNext Ultra II DNA Library Prep Kit, and paired‐end sequencing was performed on the Illumina MiSeq PE250 platform (Illumina, USA). The raw sequencing reads were deposited in the National Center for Biotechnology Information (NCBI) Sequence Read Archive (SRA) under the accession number PRJNA1378808.

Raw sequencing data were processed based on the QIIME pipeline and Caporaso guidelines, as described in previous work. Quality control was performed using Fastp with a sliding window approach (‐W4‐M20), and primer sequences were removed with Cutadapt. Operational taxonomic units (OTUs) were clustered at 97% similarity using the UPARSE algorithm, and the OTU table was rarefied to 25,487 sequences per sample. Taxa with relative abundance below 0.1% were grouped as“others”. Then, microbial community data were subsequently analyzed in terms of diversity and relative taxonomic abundance [27]. Functional prediction of KEGG orthologs (KOs) from 16S rRNA amplicon data was performed using PICRUSt2 v2.5.2 with the KEGG database (release 2023). Predicted KO abundances were normalized by 16S rRNA copy number and are reported as relative abundances [27]. These predictions represent potential functional capacity rather than actual gene expression.

### 4.5 Data Treatment and Statistical Significance Testing

All experimental data were sorted in Microsoft Excel as shown in SI‐II. Subsequent data visualization was performed using OriginPro 2021 and R 4.5.1. All errors are expressed as standard deviations (SD). For comparisons involving only two groups, two‐sample t‐tests (Welch's correction for unequal variances) were applied. For multi‐group comparisons, we employed one‐way or two‐way analysis of variance (ANOVA) followed by post‐hoc multiple comparisons with Benjamini‐Hochberg false discovery rate (FDR) correction. When assumptions of normality or homoscedasticity were violated, we used the non‐parametric Kruskal‐Wallis test followed by Dunn's post‐hoc test with FDR adjustment. All statistical analyses were performed in R version 4.5.1. No generative artificial intelligence (AI) was used in any part of this study, including experimental design, data collection, data analysis, interpretation, or manuscript preparation.

## Author Contributions

H.L., M.B., Y.W., and L.C. designed and supervised the project. H.L. performed most of the experiments and data analysis, with Z.P. assisting in the investigation. H.L. wrote the original draft, and all authors reviewed and edited the manuscript. Y.W., L.C., and M.B. acquired funding and administered the project.

## Conflicts of Interest

The authors declare no conflicts of interest.

## Supporting information




**Supporting File 1**: advs76789‐sup‐0001‐SuppMat.docx.

**Supporting File 2**: advs76789‐sup‐0002‐data.zip.

## Data Availability

The data that supports the findings of this study are available in the supplementary material of this article.
