# Supplementary material for: The Plant‐to‐Plant Circular Strategy: Coupling Photodegradation and Phytoremediation With Plant‐Based Nanomaterials for Plastic Degradation
Source: Adv Sci (Weinh). 2026 Jul 24:e76789. Online ahead of print. doi: 10.1002/advs.76789 (PMC13398134; doi:10.1002/advs.76789)
Supplement: Supplementary file 1 — Supporting File 1: advs76789‐sup‐0001‐SuppMat.docx. [file ADVS-9999-e76789-s002.docx]

**SUPPLEMENTARY INFORMATION**

Haoran Liu ^a^*, Lena Ciric ^a^, Yuheng Wang ^b^, Ziru Pei ^b^, Manpreet Bhatti ^a^*

^a^ UCL Department of Civil, Environmental and Geomatic Engineering, London, WC1E 6BT, UK

^b^ Shaanxi Key Laboratory of Qinling Ecological Intelligent Monitoring and Protection, School of Ecology and Environment, Northwestern Polytechnical University, Xi’an 710129, PR China

*These are corresponding authors

**Section S1 Plastic photodegradation experiment**

The Brunauer-Emmett-Teller (BET) analysis results of three nanomaterials (NMs) are summarized in Table S1. The phenomenon discussed in the main text Section 3.1 leads to a markedly enlarged specific surface area of the Plant-based nanomaterials (PB-NMs) (846.33 m^2^ g^-1^), which is substantially greater than that of multi-walled carbon nanotubes (MWCNTs, abbreviated as CNTs hereafter) (89.77 m^2^ g^-1^) and few-layer graphene oxide (FGO) (63.60 m^2^ g^-1^). This observation is in line with previous reports, showing that PB-NMs typically exhibit unusually high surface areas owing to their hierarchical porous architectures and abundant micro- and mesopores formed during carbonization and activation processes ^[1, 2]^. Such enlarged surface areas are generally regarded as characteristic features of biomass-derived carbon frameworks and provide a favorable basis for surface functionalization and enhanced reactivity ^[3, 4]^. However, the values of CNTs and FGO differ from those stated in the official manual, indicating that during the usage process, CNTs and FGO have undergone significant agglomeration. This is consistent with the speculation in this research that the degradation efficiency promoted by CNTs and FGO is affected by agglomeration, especially the apparent phenomenon shown in the SEM in Figure 2.

Based on Figure 2, BET data further revealed differences in the degradation pathways of the three NMs. For CNTs and GO, the specific surface area decreased, which can be attributed to the adsorption of plastic fragments within their porous structures, leading to pore blockage and reduction in accessible areas ^[5, 6]^. PB-NMs also exhibited a decrease in specific surface area, which may be explained not only by pore filling with plastic fragments, but also by partial participation of the material itself in the degradation reaction, resulting in damage to its surface structure ^[7]^.

**Table S1 The specific surface areas of the three NMs before and after the 28-day photodegradation reaction were determined by BET**

| BET Specific surface area (m^2^/g) | MWCNTs | FGO | PB-NMs |
| --- | --- | --- | --- |
| Data from manual | 100-400 | 110-250 | - |
| Before UV degradation | 89.77 | 63.60 | 846.33 |
| After UV degradation | 36.96 | 0.85 | 197.06 |


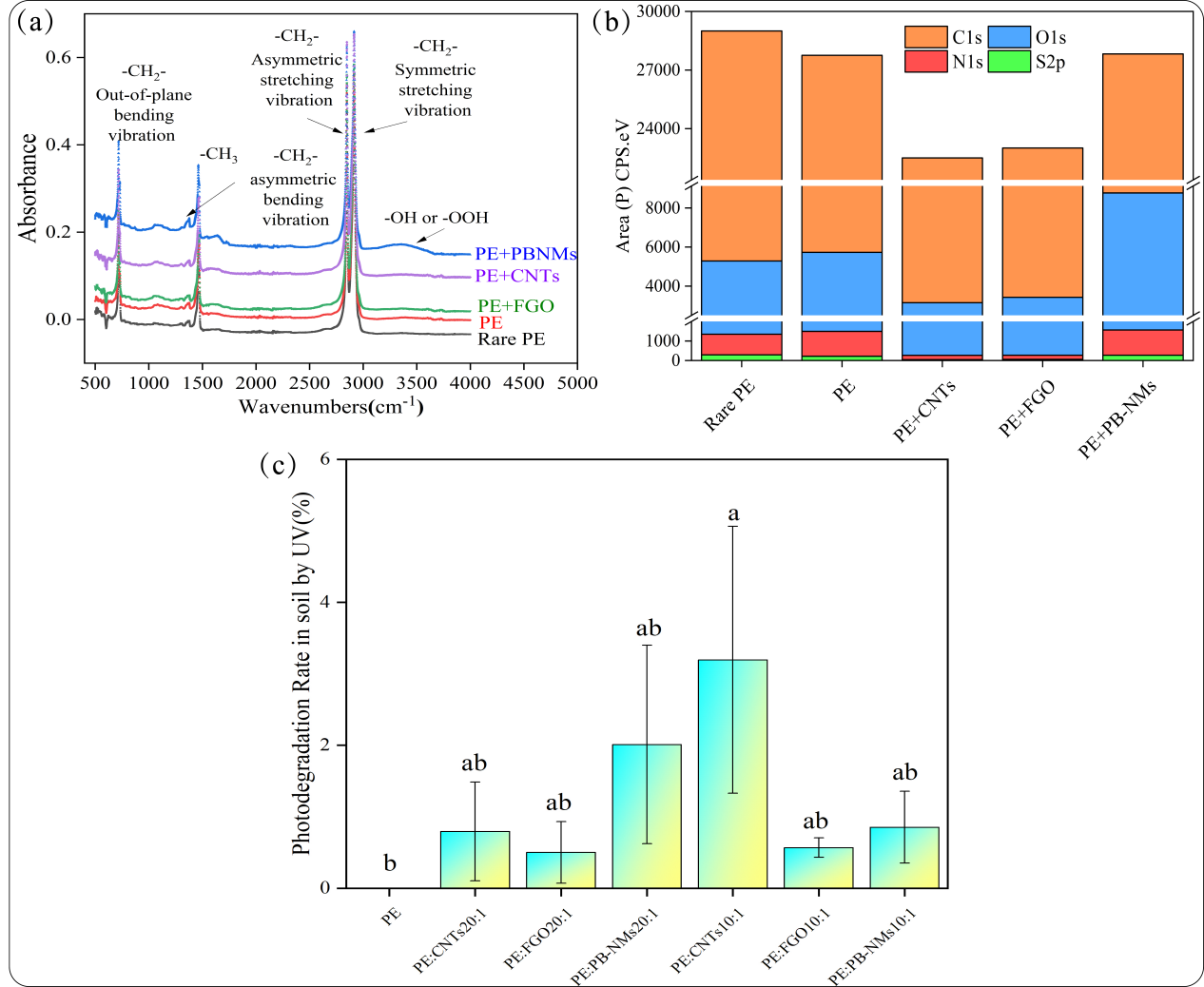


**Figure S1. (a) FT-IR and (b) XPS results of original and 28th d PE films with different NMs; the samples of the experimental group were only from PE: NMs = 20:1; (c) UV-A photodegradation of PE plastic film catalyzed by NMs, in the soil covering state, 28 d, including all experimental groups (PE:NMs=20:1 and 10:1).**

As illustrated in Figure 2(d), the Fourier-transform infrared spectroscopy (FT-IR) of CNTs and FGO reveal relatively simple surface chemistries. The stretching vibrations of C–O–C and C–O groups are located primarily in the 1050–1250 cm⁻^1^ region, while the C=O stretching vibrations characteristic of carboxyl, ester, or ketone functionalities appear more distinctly in the 1650–1750 cm⁻^1^ range. The C–H stretching bands of aliphatic and aromatic groups are observed between 2850 and 3100 cm⁻^1^, whereas the O–H stretching vibration emerges as a broad absorption centered around 3200–3400 cm⁻^1^. In contrast, the PB-NMs exhibit a considerably richer set of functional group signatures, most notably the distinct presence of –NO_2_ vibrations, which are absent in CNTs and FGO. The peak intensities of these functionalities in PB-NMs are also significantly higher, indicating a greater density and stronger contribution of surface functional groups than the other two NMs^14^.

As shown in Figure S1(a), FT-IR was employed to characterize the chemical structural evolution of polyethylene (PE) before and after photo-aging with different NMs. To thoroughly verify the effectiveness of PB-NMs and take into account the economic considerations for future applications, PE:NMs=20:1 group was selected, which was the ratio set that PB-NMs exhibited the best photodegradation-promoting effect. The pristine unaged Rare PE film exhibits the lowest overall absorbance across the full wavenumber range from 400 to 4000 cm^-1^, showing typical intrinsic characteristic peaks of neat polyethylene: the bending vibration of -CH_2_- at ~720 cm^-1^, the bending absorption of aliphatic -CH_2_/-CH_3_ at approximately 1460 cm^-1^, and the dominant asymmetric and symmetric stretching vibrations of saturated C-H at 2920 cm^-1^ and 2850 cm^-1^, respectively. After identical photo-irradiation treatment, all PE samples loaded with NMs display elevated IR absorbance accompanied by obvious baseline uplift within the 3000-3600 cm^-1^ region assigned to hydroxyl (-OH) and hydroperoxide (-OOH) groups. This suggests the initiation of photo-oxidative surface reactions triggered by NMs ^[8]^. Among all modified composites, PE+CNTs show the most pronounced increase in IR peak intensities. However, FT-IR absorbance alone does not directly quantify degradation efficiency. The gravimetric data (Figure 2a) indicate that PB-NMs achieved the highest mass loss (16.09%), followed by CNTs (9.94%) and FGO (6.79%). The strong IR signal in CNTs may reflect surface roughening rather than deeper oxidation. The blank photo-aged PE sample shows marginal absorbance increment relative to the original Rare PE, indicating slow spontaneous photo-oxidation of pure PE under illumination without catalytic NMs.

To further quantify the surface chemical composition changes and verify the formation of oxygen-containing functional groups during photo-aging, X-ray photo-electron spectroscopy (XPS) was performed, and the results are presented in Figure S1(b). All samples exhibited dominant C1s signals corresponding to the carbon backbone of PE, consistent with the characteristic hydrocarbon vibrations observed in FT-IR spectra. Compared with the initial Rare PE, the photo-aged pure PE sample showed only a slight increase in the O1s peak area, which is in good agreement with the marginal absorbance enhancement of -OH/-OOH groups in FT-IR, confirming the slow spontaneous photo-oxidation of neat PE under illumination. In sharp contrast, the three NM-loaded PE composites all displayed significantly elevated O1s peak areas, directly proving the accelerated photo-oxidation induced by the photo-catalytic NMs. Notably, the PE+PB-NMs composite showed the most pronounced increase in O1s signal intensity, indicating the highest oxygen content on its surface. This finding corroborates the FT-IR results, where PE+PB-NMs exhibited the most prominent -OH/-OOH absorption. For PE+CNTs and PE+FGO composites, their O1s peak areas were moderately increased, with PE+CNTs showing a slightly higher oxygen content than PE+FGO, which aligns with the relative absorbance enhancement of oxygen-containing groups in the FT-IR analysis. The weak N1s and S2p signals detected in the PE+PB-NMs sample further confirm the presence of residual or interacting PB-NMs on the PE surface, which may contribute to its superior photo-catalytic activity^[9]^.

Notably, the PE+PB-NMs composite shows the highest O1s peak area, indicating the greatest surface oxygen content. This appears different from the FT-IR trend, where CNTs gave the strongest absorbance. The discrepancy can be explained by the different probing depths: XPS (top ~10 nm) is more sensitive to surface oxidation, while FT-IR (several μm) is dominated by bulk signals and is also influenced by surface scattering. Thus, PB-NMs likely promote surface-limited oxidation more effectively, whereas CNTs cause greater physical roughening that enhances IR scattering.

Figure S1(c) presents the data from the soil-covered photodegradation experiment of plastic films over 28 days. 0.1 g of air-dried and sieved (200 mesh) horticultural soil was used per sample. The soil sample was evenly applied to the plastic film surface, leaving adequate gaps to allow UV light exposure. The rest of the settings were the same as those in the non-soil photodegradation experiment. From 0-21 days, no degradation above 0.5% was observed. At 28 days, only the PE:PB-NMs = 20:1 and PE:CNTs = 10:1 group showed degradation rates above 2%. This trend is similar to the uncovered experiment (Figure 2), but due to the low degradation rates and high error, the results are provided for reference only. These findings indicate that under natural conditions, such as when dust or a thin soil layer covers the surface, the UV-A and NMs degradation system cannot achieve effective degradation of plastic films. This suggests that the system is sensitive to surface cleanliness, implying that surface cleaning of plastics may be necessary in future industrial applications^[10, 11]^. However, current research still shows that photodegradation influences the degradation of fibrous microplastics in soil and further promotes the growth of microorganisms and plants ^[12]^. Therefore, the poor photodegradation effect in this study may be related to the fact that the plastic remains in a plastic film state.

**
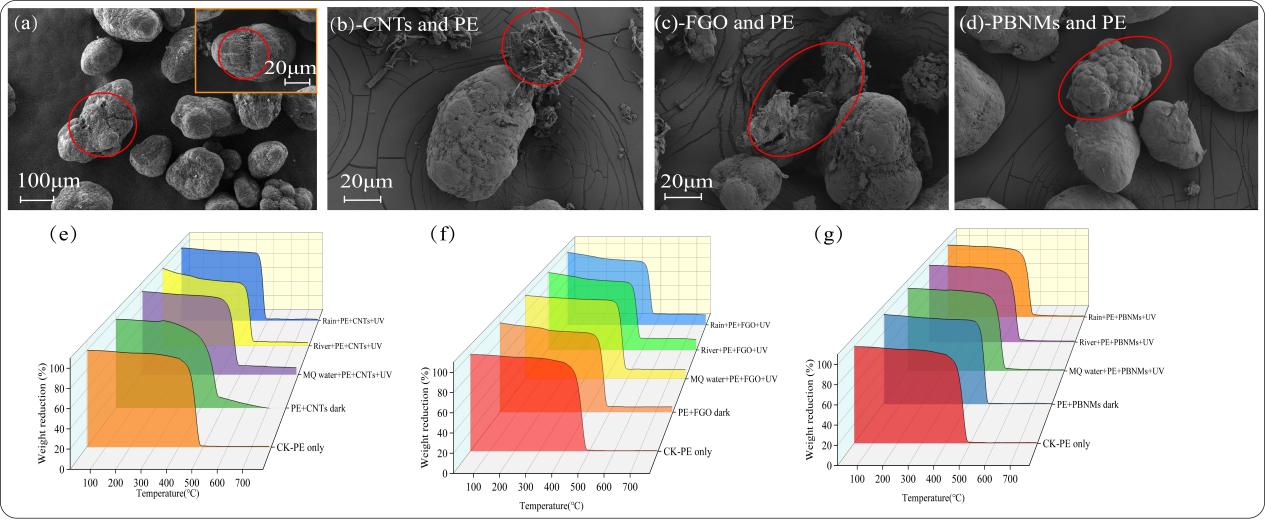
**

**Figure S2. Scanning electron microscope (SEM) of (a) Surface cracks of microplastics catalyzed by NMs; (b-d) dark control group of (b) CNTs and PE; (c) FGO and PE; (d) PB-NMs and PE.**

**Thermogravimetric analysis (TGA) of three mixtures of NMs and PE microplastic particles, including (e) CNTs and PE; (f) FGO and PE; (g) PB-NMs and PE.**

**Table S2. The comparison between the actual reaction temperature and the reference temperature.**

| Material | Tempertature range | Reference Tempertature | References |
| --- | --- | --- | --- |
| PE-Rare | 420-486℃ | 500-600℃ | ^[13, 14]^ |
| PE-Treated | 446-506℃ | 500-600℃ |  |
| CNTs in MQ | 626-800℃ | >600℃ | ^[15, 16]^ |
| FGO in MQ | 336-395℃ | 300-400℃ | ^[17, 18]^ |
| PB-NMs in MQ | 284-412℃ | <300℃ | ^[19-21]^ |

Figure S2(a) shows surface damage to MPs after joint treatment with UV-A and NMs. The large and inset images are taken from the CNTs and PB-NMs treatment groups, respectively. The observed surface ruptures are similar to those seen in the plastic film degradation experiments (Figure 2c–e).

Figures S2(b-d) present the relationships between the three types of NMs and PE particles after 28 days in the dark control. Unlike the irradiated groups (Figure 2 i-l), the control samples show clear aggregation of the NMs (marked by red circles in the panels). In these dark controls, the NMs are not well dispersed and therefore do not achieve extensive contact with the MP surfaces. Thus, it is possibility that light both promoted MP degradation and enhanced the dispersion of NMs in water ^[22, 23]^.

Figure S2(e-g) further illustrates the possible components in the PE-NMs mixtures as revealed by TGA. In Figure S2(g), the curves from the three water samples are similar, indicating that potential fine particles in the water did not exert a significant influence on the TGA process, which is consistent with the phenomenon observed in Figure 2(h). According to the PE control (CK-PE only) and the dark control (CK-PE+NMs), three main findings can be summarized. Firstly, the PE peaks in the treatment groups clearly shifted toward higher temperature regions. This shift likely results from the preferential catalytic degradation of plastic fractions with lower sublimation points by the NMs, which in turn increases the overall sublimation temperature of PE. Secondly, in the UV-A treated groups, the CNTs and FGO curves displayed distinct fluctuations below the common pyrolysis range of PE (<400°C), whereas the PB-NMs group showed only minor deviations at low temperatures, with consistent trends across the three tested water types. This indicates that while all NMs promote the breakdown of microplastics into more volatile components, PB-NMs are less sensitive to water matrix variations. Thirdly, at temperatures above the common pyrolysis range of PE (>600°C), only the CNTs and FGO groups exhibited higher residual masses than their controls, with the CNTs group retaining more than 10%. This suggests the formation of highly stable, persistent carbonaceous structures that are resistant to further degradation ^[22, 24]^. Therefore, the PB-NMs and UV-A degradation system demonstrates greater stability and application potential compared to CNTs and FGO, while also minimizing the risk of secondary pollution.

**Section S2 NM-driven phytoremediation of soil PE films**


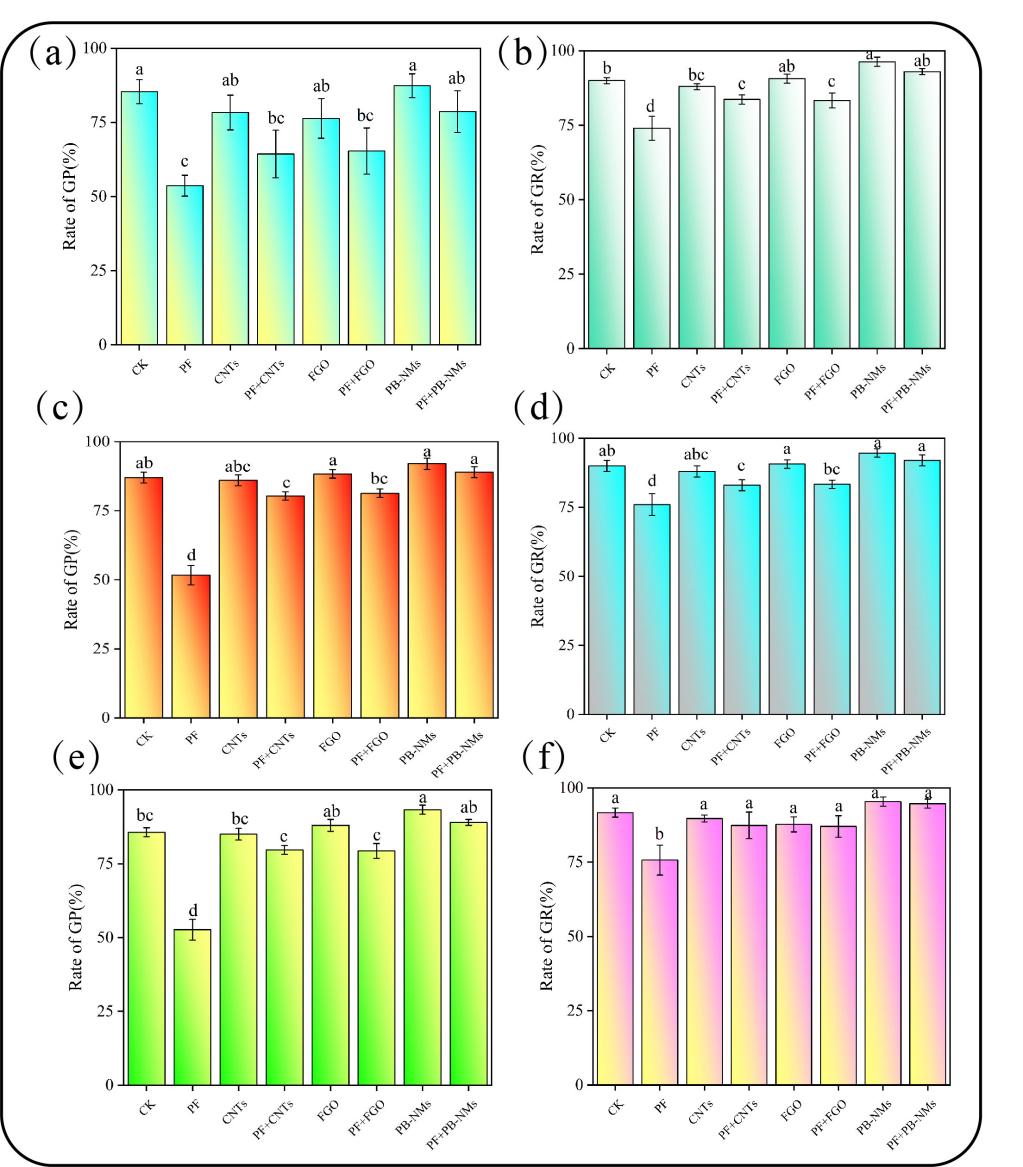


**Figure S3. Germination potential (GP, 3 days) and germination rate (GR, 7 days) for three NMs and PF combinations. The NM concentration in (a)-(b) is 200 mg/L, in (c)-(d) it is 150 mg/L, and in (e)-(f) it is 100 mg/L. The PF weight is 0.1 ± 0.005 g.**

Figure S3 shows the effects of three NMs and PE, applied alone or in combination, on the germination of Alfalfa (*Medicago sativa*) seeds. Two indicators were used: GP (3d) and GR (7d) ^[25, 26]^. When PB-NMs were applied alone, they significantly promoted GP at 100 mg/kg and significantly promoted GR at 200 mg/kg, compared with the blank control (CK). In all cases, values were higher than CK, indicating that PB-NMs themselves do not have strong phytotoxicity. In contrast, CNTs and FGO showed values slightly lower and slightly higher than CK, respectively, suggesting possible phytotoxicity or growth-promoting ability, but not significant. After PE film (PF) addition, both GP and GR were significantly lower than CK, showing high phytotoxicity of PE ^[26]^. However, in the PE+NMs groups, GP and GR were generally higher than PF, demonstrating that all three NMs could effectively mitigate the phytotoxicity of PE. Among them, PB-NMs showed the strongest effect, with GP and GR values exceeding CK at all tested concentrations. These results indicate that PB-NMs have better plant protection ability than CNTs and FGO and can promote Alfalfa germination in the 100-200 mg/kg range.


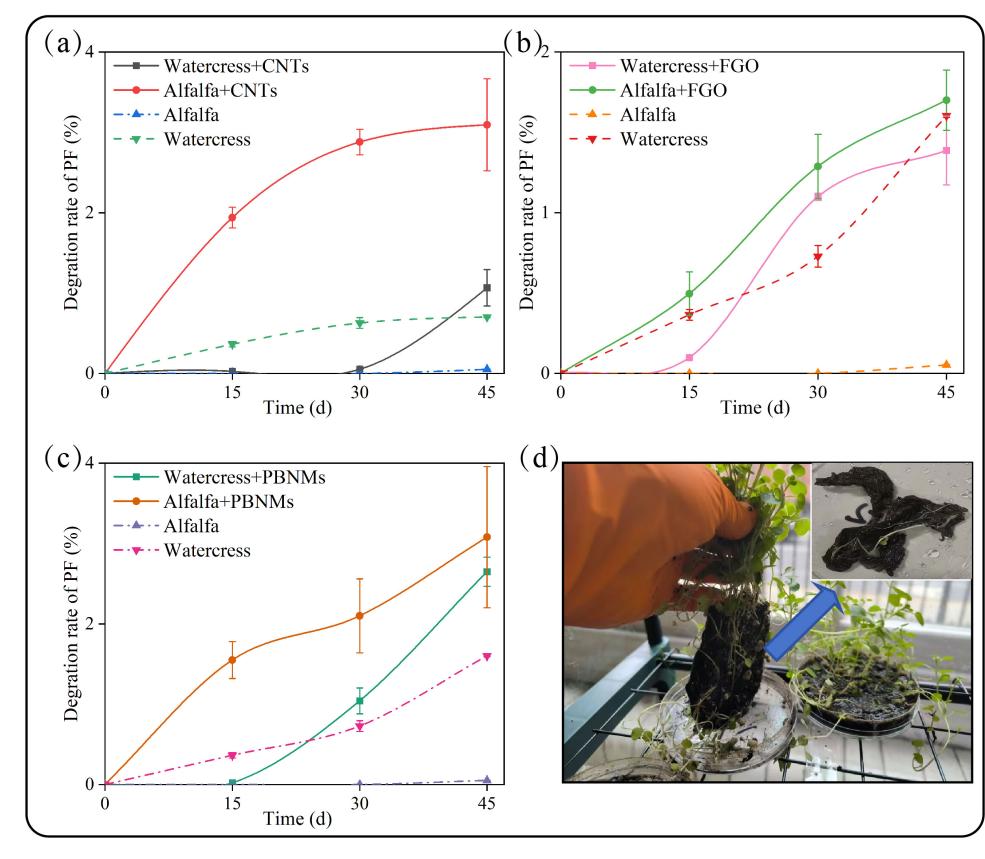


**Figure S4. Preliminary experiments on plastic film degradation in hydroponic soil covering of alfalfa and watercress, assisted by NMs. (a) 1 mL 100 mg/L MWCNTs group; (b) 1 mL 100 mg/L FGO group; (c) 1 mL 100 mg/L PB-NMs group; (d) Schematic diagram at the end of 45 days of incubation, showing the entanglement between the roots and the plastic film. The amount of soil added to each sample was 10 g. The PF weight is 0.1 ± 0.005 g.**

Figure S4 shows the results of the pre-experiment of cultivation. The setup is shown in Figure S4(d). The combination of alfalfa with the three NMs showed higher degradation rates than watercress with NMs. In terms of trends, with plant growth, CNTs did not further enhance plastic film degradation, while the PB-NMs group still showed potential for higher degradation rates. The effect of FGO was not significantly different from the control. This pattern was similar to the degradation results observed in the pot experiment shown in Figure 3. Since the 100 mg/kg concentration used in the pre-experiment showed weak effects, the formal experiment was designed with a concentration range of 100-200 mg/kg. Because Alfalfa had better degradation performance than watercress, and as a legume has been widely reported for degradation of soil organic pollutants ^[27]^, while watercress as a hydrophilic plant has limited growth capacity in soil, Alfalfa was finally chosen as the plant for phytoremediation.

Figure S4(d) also shows the wrapping and “folding” of the plastic film by plant roots during the reaction process, which explains why, after 90 d of cultivation in the formal experiment, the roots of Alfalfa could reach the bottom of those pots without piercing the plastic film on the surface.


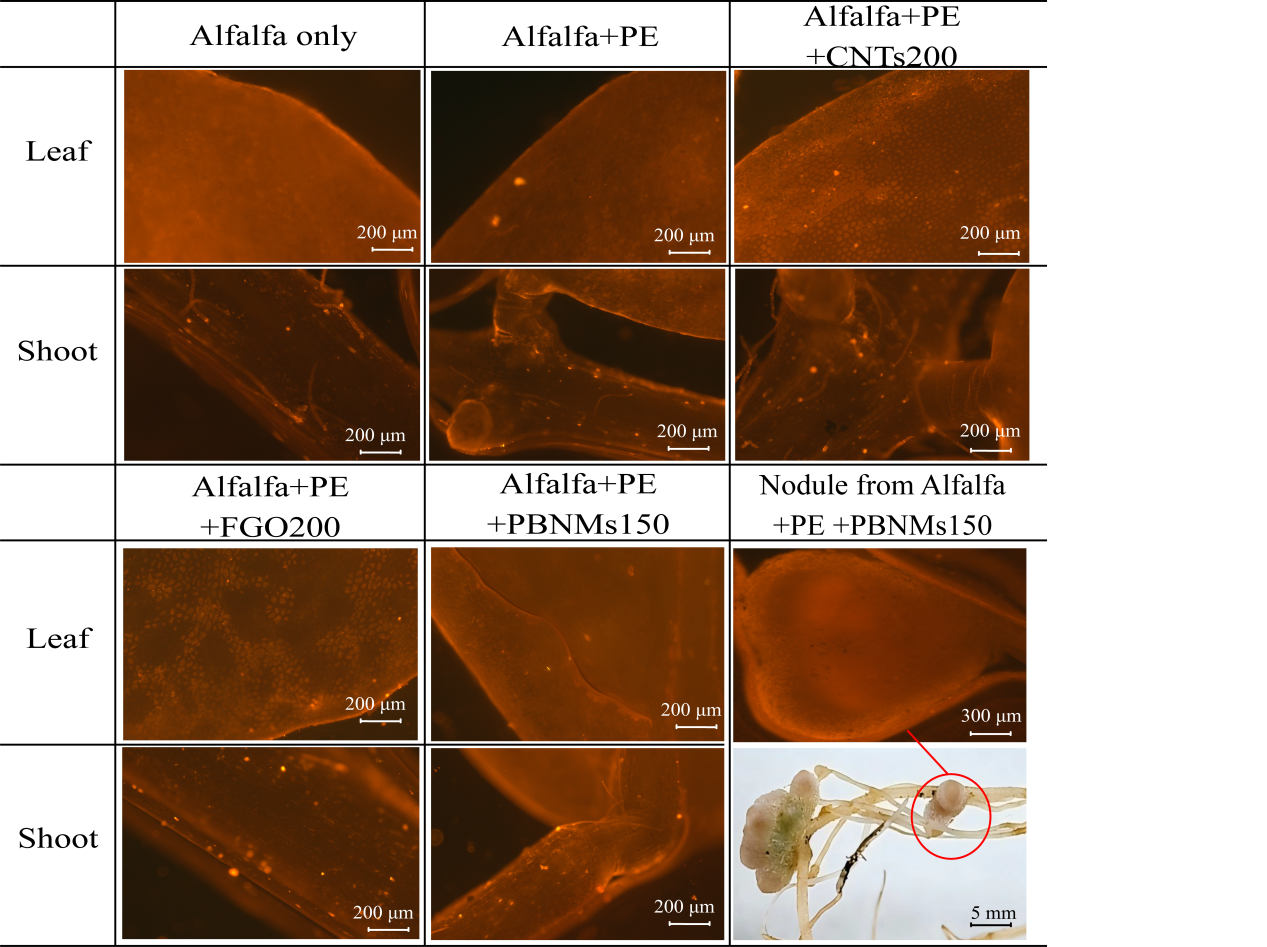


**Figure S5. Fluorescence test of plant leaves, shoots and root nodules, stained with Nile red for 10 minutes.**

Figure S5 presents the fluorescence micrographs of PE within plant tissues other than the roots. In the Alfalfa only group, no bright fluorescent signals were detected in the leaves; however, fine bright spots were observed in the shoots. These spots were identified as stomata and emerging lateral buds, whose structures were clearly visible under higher magnification. In the groups with added PE, a small number of bright spots appeared in the leaves. Upon magnification, and based on their distribution within mesophyll cells, it could not be ruled out that these might be stained chloroplasts. Additionally, some bright spots located clearly outside the plant tissue were attributed to incompletely rinsed dye residues, as seen in the images of Alfalfa+PE and Alfalfa+PE+CNTs200. No prominent bright patches were found in the stained root nodules. These observations suggest that PE may not have entered the shoots^[28]^. Given the presence of autofluorescent structures and Nile red-affinity components in shoots and leaves, this hypothesis requires further verification.


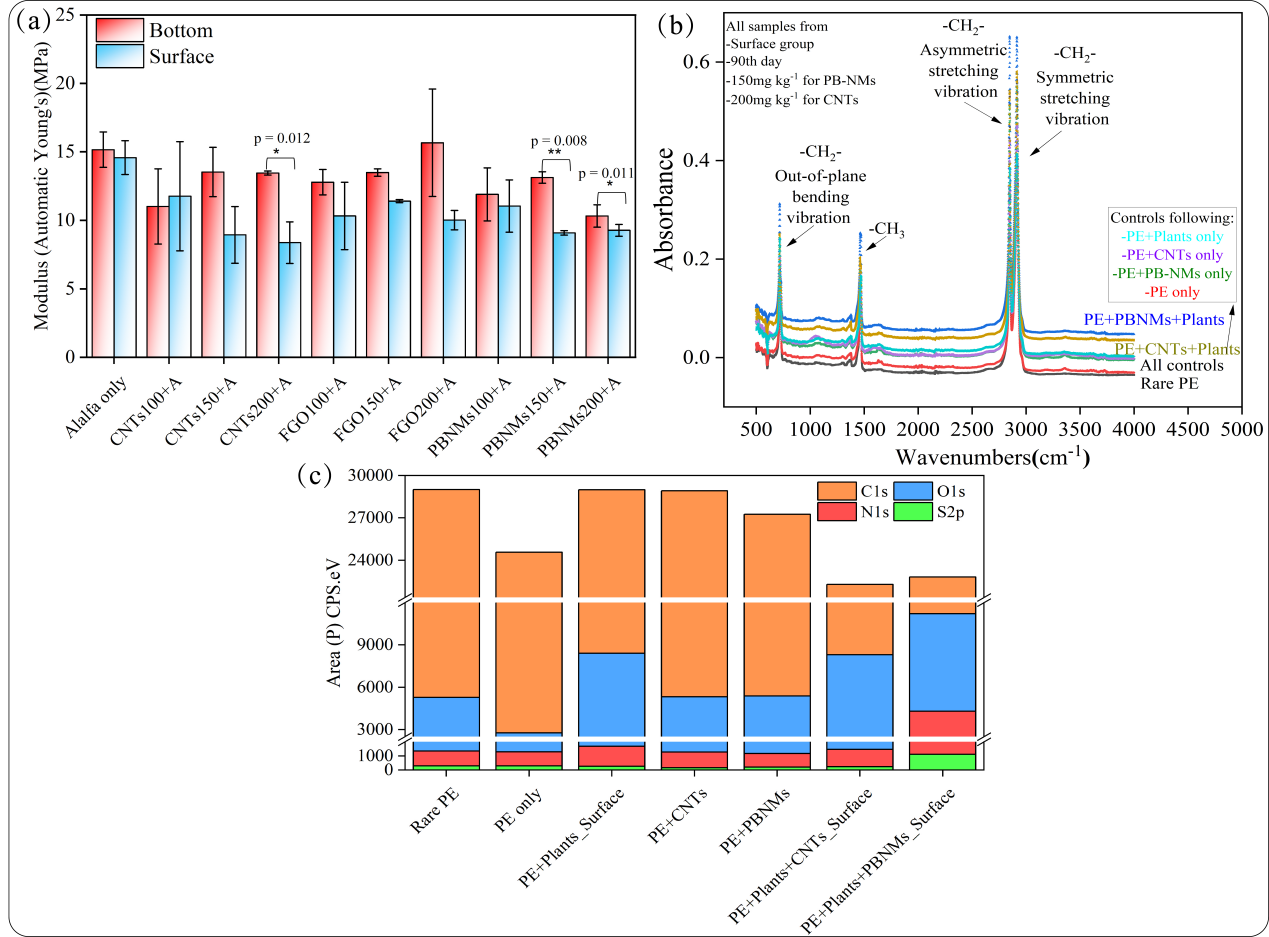


**Figure S6. (a) The tensile strength of plastic film in the pot experiment (expressed by Young's modulus, MPa); (b) FT-IR spectra of PE films after 90 days of phytoremediation with PB-NMs150_Surface and CNTs200_Surface; (c) XPS elemental analysis of corresponding PE film surfaces.**

**Table S3 Control groups of the tensile strength of plastic film in the pot experiment**

| Groups | Young's modulus（MPa） | Sample quantity |
| --- | --- | --- |
| Rare-PF | 782.79±0.26 | 3 |
| Blank-PF only | 523.77±25.11 | 3 |
| Control-NMs+PF only | 499.01±63.89 | 27 |

Figure S6(a) and Table S3 show the strength of plastic films after 90 d of the pot experiment. In all plant groups, film strength was less than 2% of the original plastic, lower than both the Blank control-PF only and the Control-NMs+PF only (about 5%). This indicates that plants significantly promoted the reduction of film strength ^[29, 30]^. Among the experimental groups, CNTs200, PB-NMs150, and PB-NMs200 showed significant differences between surface and bottom films, which corresponded to the groups with the highest degradation rates in Figure 3. Other groups also generally showed weaker surface strength than bottom strength, further demonstrating the major role of plant growth in reducing plastic film strength. Even for CNTs, which did not show plant dependence in degradation rate, plant presence was still required to reduce film strength and accelerate degradation.

As shown in Figure S6(b), FTIR was performed to examine the surface chemical changes in PE films after 90 days of phytoremediation, with and without the addition of PB-NMs and CNTs. The two experimental groups with the highest degradation rates (PB-NMs 150_Surface and CNTs200_Surface) were analyzed to demonstrate the possible differences in the promoting degradation of two NMs.

The unaged Rare PE control shows the lowest absorbance across the entire wavenumber range, displaying the characteristic intrinsic peaks of polyethylene: the out-of-plane bending vibration of -CH_2_- at ~720 cm^-1^, the bending vibration of -CH_3_ at ~1460 cm^-1^, and the asymmetric and symmetric stretching vibrations of -CH_2_- at ~2920 cm^-1^ and ~2850 cm^-1^, respectively. Among all treated samples, PE films exposed to both plants and NMs (PE+PBNMs+Plants and PE+CNTs+Plants) exhibit significantly higher absorbance at these PE-related peaks than the control groups, suggesting that plant-mediated processes, in combination with NMs, lead to increased surface roughness or structural changes in the PE matrix. Specifically, the absorbance intensity of PE+PBNMs+Plants is comparable to that of PE+CNTs+Plants, consistent with the similar degradation rates observed for both treatments in this study. However, when combined with plant growth and degradation rate data, the contributions of CNTs and PB-NMs appear to differ. While both treatments result in enhanced PE chain modification, the CNTs-only and PBNMs-only controls show much lower absorbance than their plant-containing counterparts, indicating that direct abiotic modification by the NMs alone is limited. Notably, the plant-only control (PE+Plants only) shows moderate absorbance enhancement, which is further increased when combined with PB-NMs, supporting the hypothesis that PB-NMs promote plant growth and rhizosphere activity, thereby indirectly amplifying plant-mediated PE degradation. In contrast, although CNTs+Plants achieve similar FTIR absorbance levels, the known phytotoxicity of CNTs suggests their contribution may stem from a different pathway, which is direct abiotic catalytic degradation rather than plant-stimulated biological activity.

To further quantify these surface chemical changes, XPS elemental analysis was shown in Figure S6(c). Consistent with the FT-IR observations, all samples were dominated by the C1s signal, confirming the retention of the PE carbon backbone ^[9]^. Compared with Rare PE and the abiotic PE-only control, the plant-only treatment showed a moderate increase in O1s peak area, indicating mild rhizosphere-induced surface oxidation. Notably, the PE+Plants+CNTs and PE+Plants+PBNMs treatments exhibited the most significant elevation in O1s intensity, with PE+Plants+PBNMs showing the highest oxygen content. This corroborates the FTIR data, where these two plant+NM combinations displayed the strongest absorbance enhancement. The abiotic NM-only controls (PE+CNTs and PE+PBNMs) showed only marginal increases in O1s signal, supporting that direct abiotic modification by the NMs alone is limited, as suggested by FT-IR. For PE+Plants+PBNMs, the significantly higher O1s content, together with detectable N1s and S2p signals, supports the degradation rate-based hypothesis that PBNMs enhance plant rhizosphere activity, indirectly amplifying biological PE degradation. In contrast, while PE+Plants+CNTs achieved comparable O1s levels, the absence of such heteroatom signals aligns with the interpretation that its contribution likely arises from direct abiotic catalytic pathways.

Importantly, no prominent carbonyl (C=O) or hydroxyl (-OH) peaks were detected in any group from FTIR spectra. Meanwhile, XPS results showed no obvious oxygen enrichment across all samples. These two findings jointly confirm that the surface modification of PE films is dominated by plant-rhizosphere interactions instead of extensive photo-oxidative degradation (Figure 2 and S1).

**Section S3 Analysis of micoorganisms in NM-driven phytoremediation**


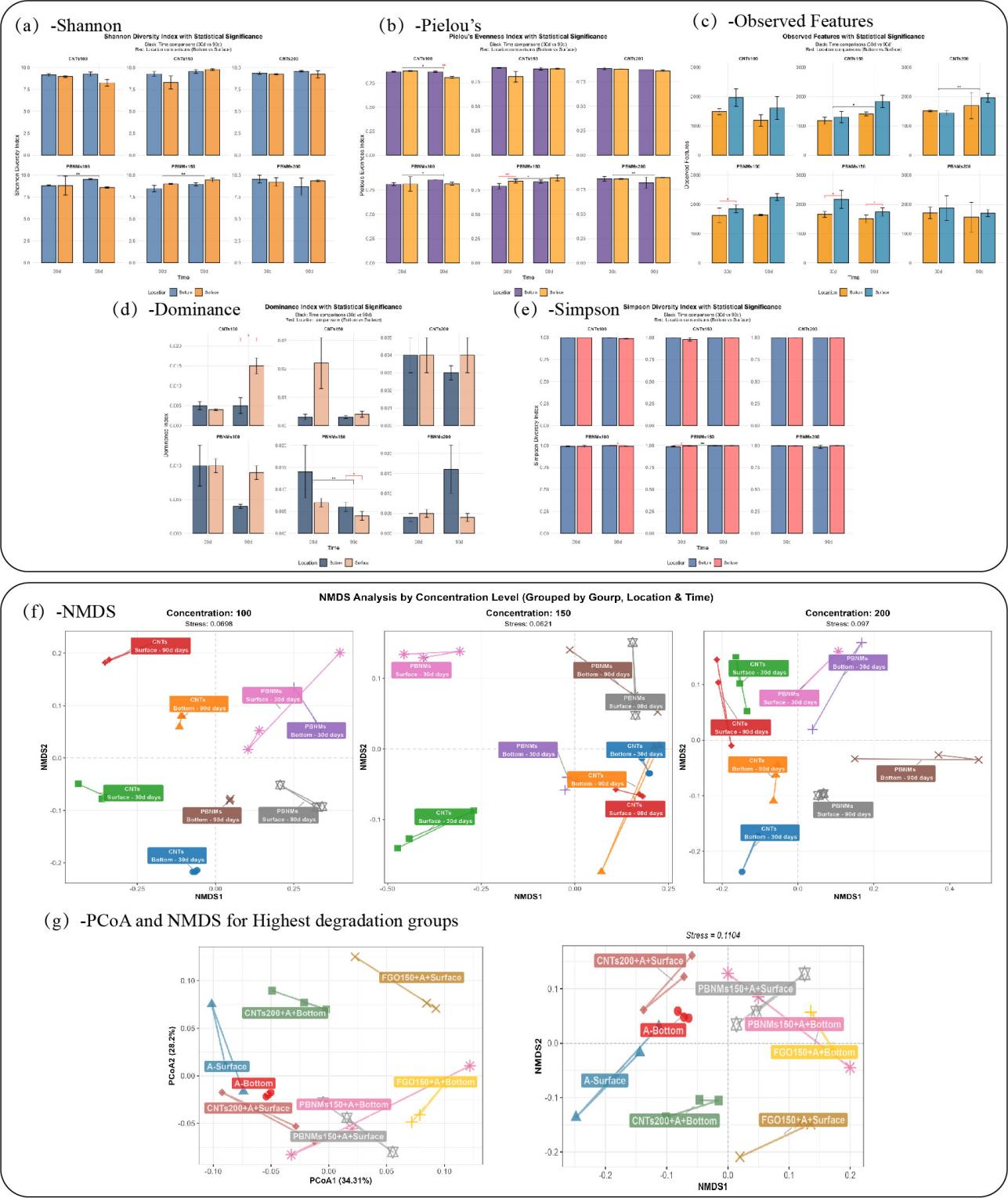


**Figure S7 Microbial community diversity, including (a-e) α diversity, including (a) Shannon; (b) Pieou's; (c) Observed Features; (d) Dominance; (e) Simpson. (f-g) β diversity, including (f) NMDS analysis for three NMs concentrations; (g) PCoA and NMDS analysis for the top three degradation groups.**

Note：The image DPI>600, can be seen clearly after enlarging.

Figure 4 presents the α diversity indices represented by Chao1, while Figure S7 shows the remaining five commonly used indices. Among them, Observed Features, as the most direct metric, clearly reflect significant temporal changes in the CNTs group and both temporal and spatial (Bottom and Surface) variations in the PB-NMs group. The Simpson index indicates that all groups remain within a high-diversity range, suggesting that the microbial communities are still in a relatively healthy state. The Dominance index, contrary to the Simpson Diversity Index (1–D), emphasizes the unevenness of species distribution; based on Simpson’s results, it further highlights the distinct community differences in the PB-NMs groups, particularly the significantly increased diversity (lower dominance) in the 90d Surface group. The Shannon index also shows high diversity across all treatments (values > 2), while revealing significant differences in the PB-NMs-treated groups. The Pielou_e index, as the normalized form of the Shannon index, more clearly demonstrates the same significant differences observed in Observed Features and Chao1. These results indicate that PB-NMs150 and Alfalfa treatments significantly enhanced community diversity.

Figure S7(f) presents the Non-metric Multidimensional Scaling (NMDS) analysis, which shows patterns similar to those observed in the PCoA analysis in Figure 4 ^[31, 32]^. Figure S7(g) compares the high-concentration degradation groups of the three NMs at 90 days within the same ordination plot. The results indicate that the Alfalfa only control group and the PB-NMs groups (Surface and Bottom) cluster closely together, while the FGO and CNTs groups are positioned farther apart. This suggests that Alfalfa exerted a stronger influence on the microbial community in the PB-NMs treatments compared with the other two NMs. These findings further support the conclusions from Figures 4-5.


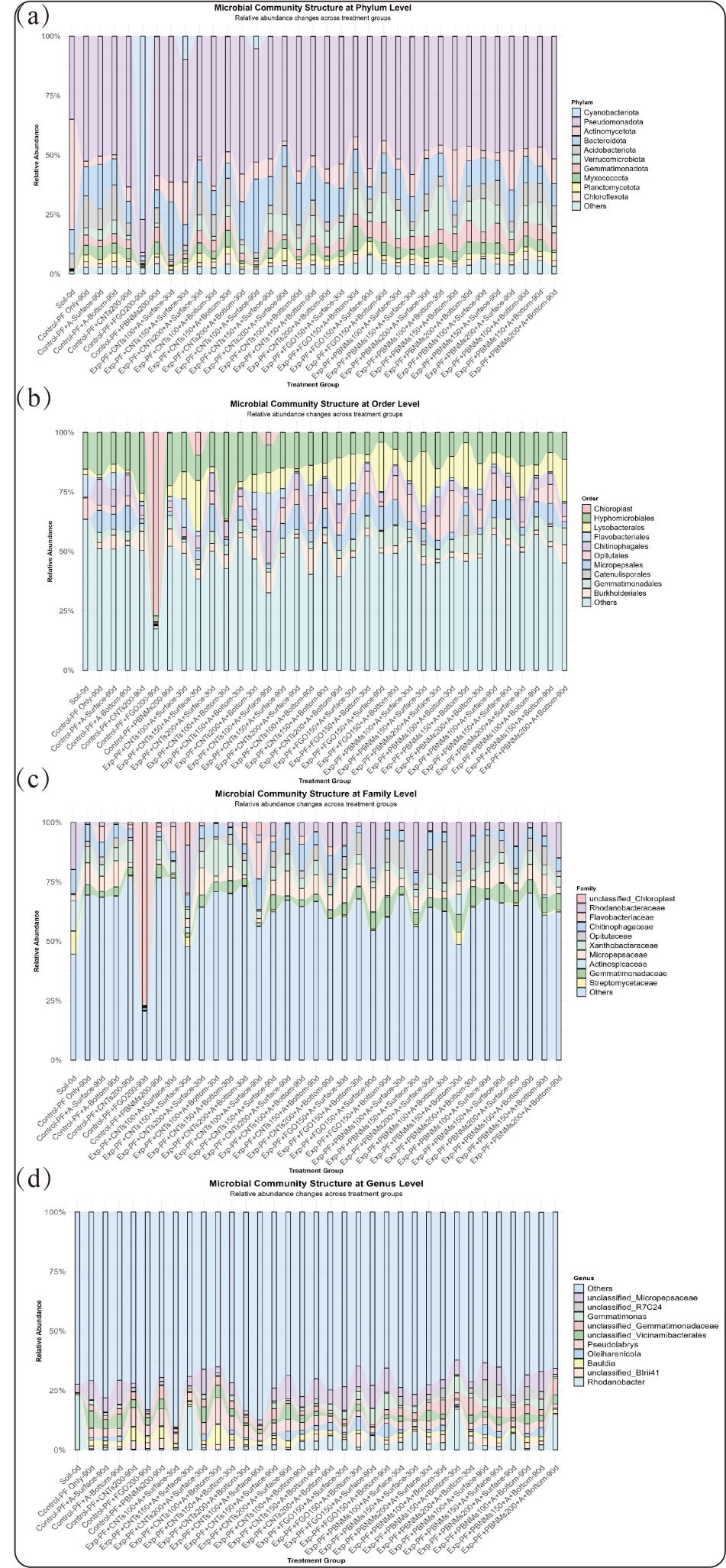


**Figure S8 TOP10 relative abundances of microbial communities, including four levels: Phylum, Order, Family, and Genus.**

Note：The image DPI>600, can be seen clearly after enlarging.

Figure S8 presents the relative abundance of microbial communities across all groups at four taxonomic levels. At the Phylum level, the high-degradation groups showed higher abundances of *Pseudomonadota*, *Verrucomicrobota*, and *Gemmatimonadota* compared with the control. At the Order level, these groups were characterized by increased abundances of *Lysobacterales* and *Opitutales*. At the Family level, higher abundances of *Rhodanobacteraceae* and *Gemmatimonadaceae* were observed in the high-degradation groups. These results are consistent with the findings discussed in Figures 4-5 and Table 1, confirming the reliability of microbial community data.

In addition, the microbial communities exhibited more distinct trends related to concentration and spatial position (Surface or Bottom). This pattern suggests that, as plant growth progresses, location-based differences in microbial communities gradually diminish, while the type and concentration of NMs continue to influence community abundance. This also explains why the degradation rate correlates with both NM type and concentration. For example, at the Phylum level within the CNTs group, *Acidobacteriota*, *Verrucomicrobiota*, and *Pseudomonadota* showed higher abundances in CNTs 200, which is the group with the highest degradation rate, indicating that CNTs 200 may promote the growth of degradative microorganisms to enhance degradation. In the PB-NMs 150 group, which is another group with the highest degradation rate, increases were mainly observed in *Acidobacteriota* and *Gemmatimonadota*. These findings support the conclusion derived from Figures 4-5 that PB-NMs and CNTs promote degradation through similar but not identical mechanisms.


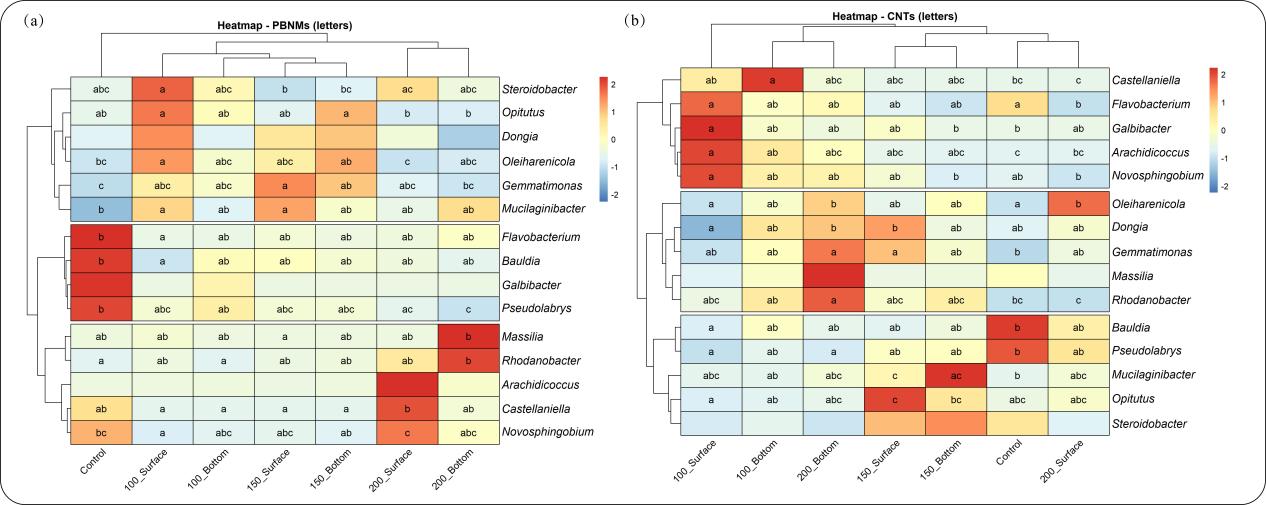


**Figure S9 Heatmap of CNTs and PB-NMs in three concentrations in 90d campared to the control (Plastic only group).**

Note: The image DPI>600, can be seen clearly after enlarging.

Figure S9 illustrates the microbial communities of PB-NMs and CNTs at 90 days across three concentration levels. A clear clustering pattern driven by the PB-NMs concentration gradient can be observed, along with distinct dominant microbial taxa at each concentration, which may explain the variation in degradation rates. Notably, Rhodanobacter, a known plastic-degrading bacterium, showed the highest abundance in the PB-NMs 200 Bottom group. Combined with the results from Figures 4 and S8, where this pattern was not observed at 30 days, this indicates that as plant roots extended to the bottom of the pots, the growth of degradative microorganisms was significantly promoted. In contrast, the clustering pattern based on concentration was not observed in the CNTs group with the highest degradation rate (CNTs 200). This suggests that the potential phytotoxic effects of high CNTs concentrations interfered with the plant’s ability to regulate the microbial community at 90 days, preventing the convergence seen in the lower-concentration groups. This confirms the findings in Figure 4.


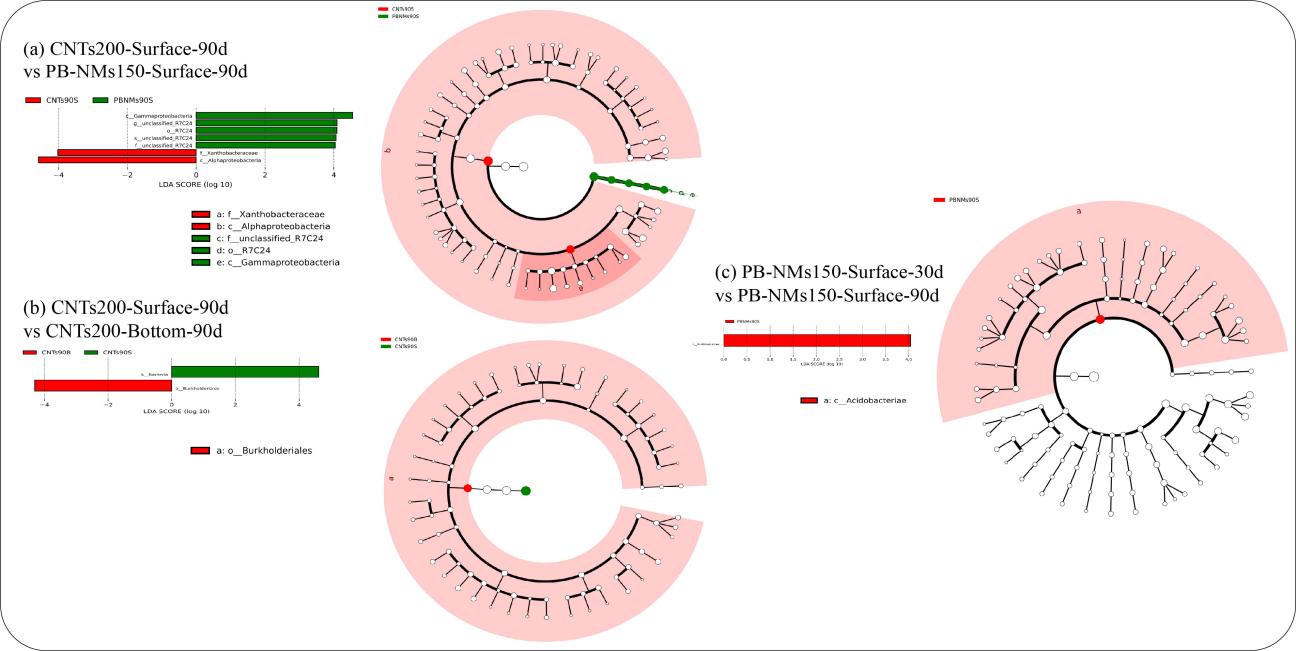


**Figure S10. LEfse analysis of (a) CNTs200 Surface 90d vs PB-NMs150 Surface 90d; (b) CNTs200 Surface 90d vs CNTs200 Bottom 90d; (c) PB-NMs150 Surface 30d vs PB-NMs150 Surface 90d.**

Figure S10 presents the results of the LEfSe (Linear Discriminant Analysis Effect Size) analysis^[33]^. The comparison between the CNTs and PB-NMs Surface groups revealed distinct differences, further confirming the presence of different enhancement mechanisms between the two NMs. The comparison between the Surface and Bottom samples within the CNTs group showed only minor differences, while no significant differences were detected within the PB-NMs group (and thus no results are displayed). Figure S10(c) compares the PB-NMs groups at 30 and 90 days, revealing only one differential microbial taxon. These results support Figure 4.


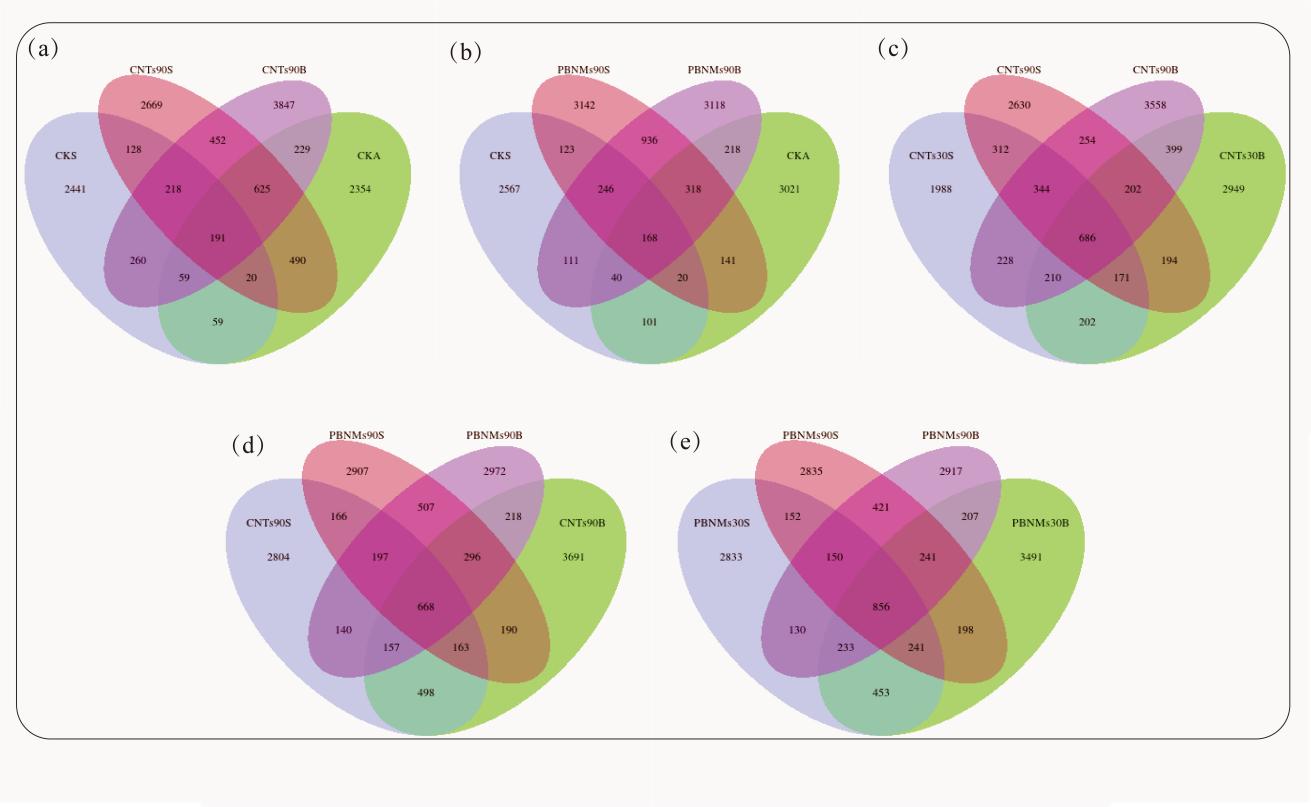


**Figure S11. Venn diagram, including (a) Soil with plastic film only 90d (CKS) vs**

**CNTs200 Surface 90d vs CNTs200 Bottom 90d vs Alfalfa+plastic only surface 90d (CKA) ; (b) CKS vs PBNMs150 Surface 90d vs PBNMs150 Bottom 90d vs CKA; (c) CNTs200 Surface 90d vs CNTs200 Bottom 90d vs CNTs200 Surface 30d vs CNTs200 Bottom 30d; (d) CNTs200 Surface 90d vs CNTs200 Bottom 90d vs PBNMs150 Surface 90d vs PBNMs150 Bottom 90d; (e) PBNMs150 Surface 90d vs PBNMs150 Bottom 90d vs PBNMs150 Surface 30d vs PBNMs150 Bottom 30d.**

Figure S11 provides a visual representation of the temporal and spatial differences among the NM treatments using Venn diagrams ^[34, 35]^. Figures S11(a–b) compare the Surface and Bottom samples of the NM-treated groups at 90 days with two control groups. The results show that, compared with CNTs, PB-NMs exhibited a greater number of unique taxa when compared to the controls. Moreover, PB-NMs displayed a higher number of shared taxa between the Surface and Bottom samples than CNTs, which are 936 and 452, respectively. Based on Figures S11(c) and (e), the number of shared taxa between 30 and 90 days was higher in the CNTs groups than in the PB-NMs groups. For instance, at the Surface level, the shared taxa numbered 312 and 152, respectively. This pattern is consistent with the previously discussed results, suggesting that the synergistic interaction between plants and PB-NMs drives community shifts distinct from those in the CNTs treatments. Figure S11(d) further shows that CNTs and PB-NMs groups shared a large number of taxa (668) at 90 days, indicating a trend toward community convergence once the degradation process reached a stable phase. These observations are consistent with the conclusions presented in Figures 4 and S9.


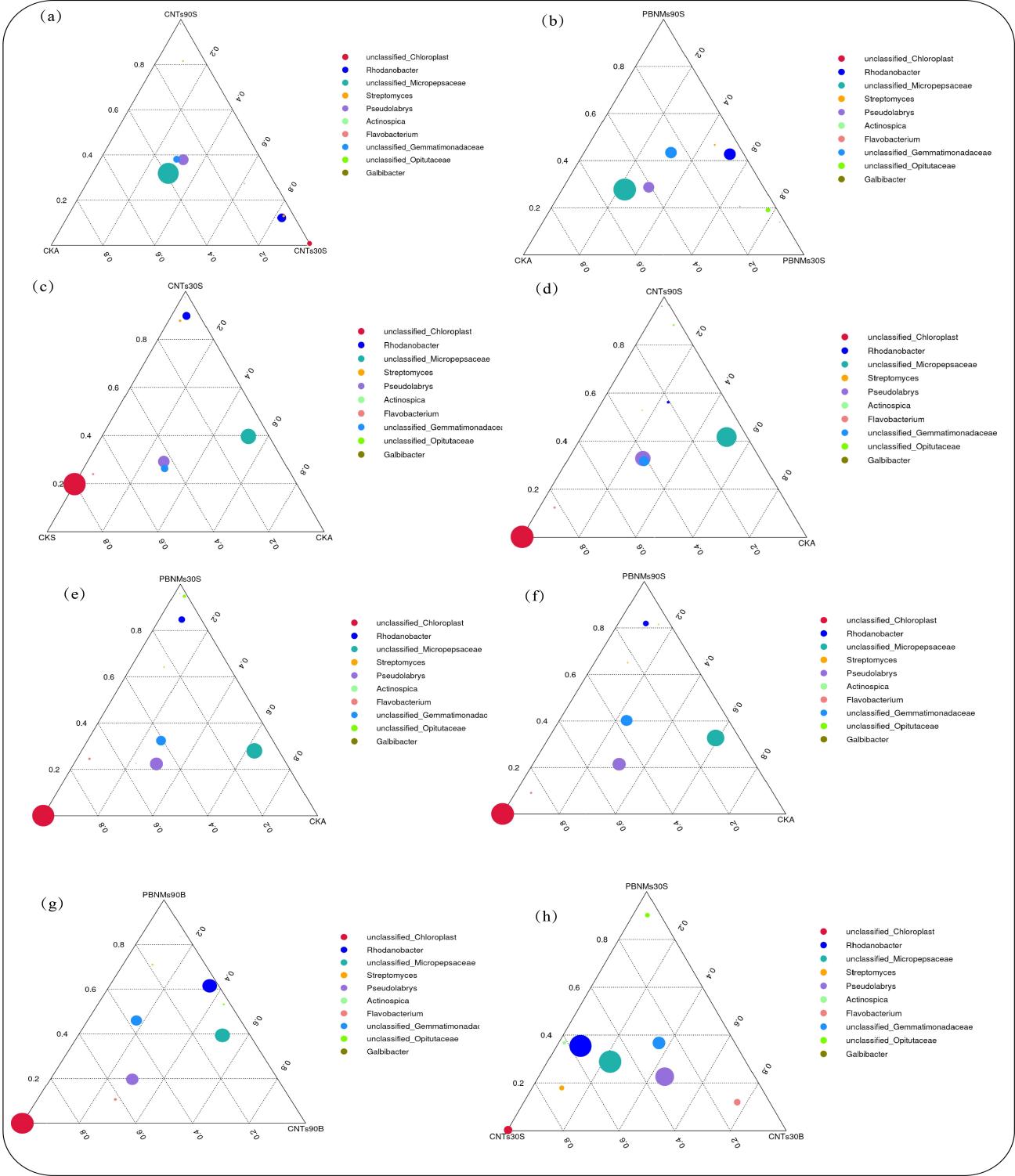


**Figure S12. Ternary diagram, including (a) Alfalfa+plastic only(CKA) vs CNTs200 in 30d surface vs CNTs200 in 90d surface;(b)CKA vs PB-NMs150 in 30d surface vs PB-NMs150 in 90d surface; (c) Soil with Plastic only (CKS) vs CKA vs CNTs200 in 30d surface; (d) CKS vs CKA vs CNTs200 in 90d surface; (e)CKS vs CKA vs PB-NMs in 30d surface; (f) CKS vs CKA vs PB-NMs in 90d surface; (g) CKS vs CNTs200 Bottom in 90d vs PB-NMs150 Bottom in 90d; (h) CNTs200 Surface 30d vs CNTs200 Bottom 30d vs PBNMs Surface 30d.**

Figure S12 uses ternary plots to validate the conclusions drawn from the Venn diagram analysis ^[36, 37]^. Notably, degradative bacteria such as Rhodanobacter were positioned closer to the CNTs 30d coordinates rather than CNTs 90d, and they were also present in both PB-NMs 30d and 90d samples. This pattern indicates that the degradation-promoting effect of PB-NMs is gradual and sustained, whereas that of CNTs is rapid but transient. These observations are consistent with the degradation trends shown in Figure 3.

**
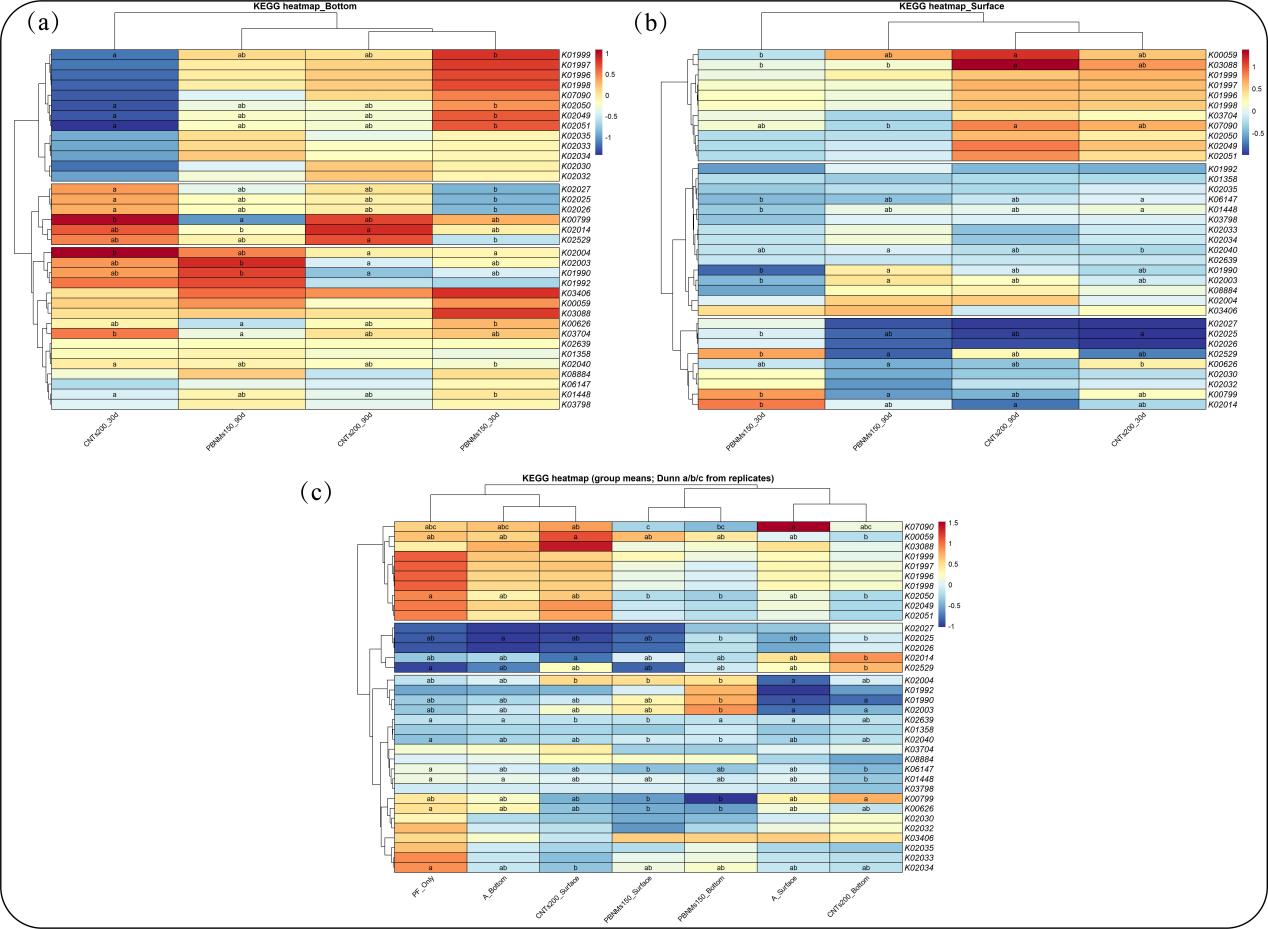
**

**Figure S13. Heatmap of KEGG functional gene, including (a) two highest degradation groups (Bottom) from 30 to 90d; (b) two highest degradation groups (Surface) from 30 to 90d; (c) comparison between two highest degradation groups and the control group at 90 days.**

Figure S13 (a-b) shows the functional gene gap of high degradation rate on the time scale. Among them, functional genes with significant advantages or disadvantages were used for the correlation analysis in Figure 5. Table S4 lists the main functions of these functional genes. The results of Figure S13(c) indicated that the results of the NMs addition group were similar only to those of the Surface group in the alfalfa control group, which was in line with the previously proposed conclusion that plant growth and NMs work together to achieve degradation.

The genomic potential for PE degradation is evidenced by the upregulation of key genes encoding specific metabolic functions^[38]^. Most critically, K00059 (3-hydroxyacyl-CoA dehydrogenase) is implicated in the β-oxidation of aliphatic chains, directly targeting the hydrocarbon backbone of PE and cleaving it into acetyl-CoA units for cellular energy production^[39]^. This initial oxidative process is likely supported by K02003 (iron complex transport system substrate-binding protein), which ensures the supply of iron, a vital cofactor for oxidative enzymes that initiate polymer breakdown. Subsequently, K00799 (glutathione S-transferase) plays a crucial role in cellular detoxification by conjugating glutathione to hydrophobic and potentially toxic intermediates generated from the incomplete oxidation of PE, enhancing their solubility and mitigating cellular stress. Finally, K07090 (putative acyl-CoA thioesterase) may further process these oxidized, fatty acid-like fragments, regenerating free CoA to sustain continuous rounds of the beta-oxidation cycle and complete the metabolic assimilation of the polymer.

**Table S4 The function of the KEGG functional gene with significant changes**

| KO ID | Function | Potential Role in Plastic Degradation | KEGG Link |
| --- | --- | --- | --- |
| **K03088** | DNA-directed RNA polymerase subunit beta | General cellular metabolism and stress response. Under stressful conditions like plastic exposure, cells may upregulate transcription to produce degradation enzymes. | [https://www.genome.jp/entry/K03088](https://www.genome.jp/entry/K03088) |
| **K01990** | Glycyl-tRNA synthetase beta subunit | Protein synthesis. Essential for producing all proteins, including enzymes involved in plastic biodegradation pathways. | [https://www.genome.jp/entry/K01990](https://www.genome.jp/entry/K01990) |
| **K00059** | 3-hydroxyacyl-CoA dehydrogenase | Fatty acid beta-oxidation. This enzyme can potentially degrade aliphatic chains in polymers like polyethylene, breaking them down into acetyl-CoA for energy. | [https://www.genome.jp/entry/K00059](https://www.genome.jp/entry/K00059) |
| **K02014** | Polyamine transport system substrate-binding protein | Uptake of polyamines. Polyamines are involved in stress responses. Plastic degradation can be a stressful process, and this transporter may help the cell cope. | [https://www.genome.jp/entry/K02014](https://www.genome.jp/entry/K02014) |
| **K02529** | ABC-2 type transport system permease protein | General polysaccharide and drug efflux. Could be involved in transporting degraded plastic oligomers or by-products out of the cell, or in importing nutrients. | [https://www.genome.jp/entry/K02529](https://www.genome.jp/entry/K02529) |
| **K02003** | Iron complex transport system substrate-binding protein | Iron uptake. Iron is a key cofactor for many oxygenases and peroxidases that are known to catalyze the oxidative cleavage of plastic polymer chains. | [https://www.genome.jp/entry/K02003](https://www.genome.jp/entry/K02003) |
| **K02040** | ABC-2 type transport system ATP-binding protein | It provides energy for transportation. Works with permease proteins (like K02529) to actively export or import molecules relevant to the degradation process. | [https://www.genome.jp/entry/K02040](https://www.genome.jp/entry/K02040) |
| **K00799** | Glutathione S-transferase | Detoxification and conjugation. Can conjugate glutathione to hydrophobic, potentially toxic intermediates generated during plastic breakdown, making them less toxic and more soluble. | [https://www.genome.jp/entry/K00799](https://www.genome.jp/entry/K00799) |
| **K07090** | Putative acyl-CoA thioesterase | Hydrolysis of acyl-CoA compounds. May process fatty acid-like intermediates derived from the oxidation of plastic polymers, regenerating CoA for further metabolic cycles. | [https://www.genome.jp/entry/K07090](https://www.genome.jp/entry/K07090) |

**Section 3 Experimental material and method details**

**Natural water sample collection method**

River water and rainwater samples were collected from the Lee Valley, London (51.59°N, 0.051°W) on January 27, 2025 as shown in Figure S14, which is the London water source area. River water (2 L) was collected at about 10-11:00am using a horizontal water sampler and immediately transferred into a pre-cleaned, narrow-mouth glass bottle. As shown in Figure S14, samples 1-3 were taken from the bridge and both sides and then mixed to avoid the influence of river flow on the water sample quality. Rainwater (about 1 L) was collected directly during a heavy rainfall (from approximately 13:00 to 15:00, spanning the onset to the end of precipitation) using five wide-mouth glass containers placed in an open area. After the collection, the water was stored at 4 °C and used within 48 hours. The water sample was filtered through a 0.45 µm membrane before use.


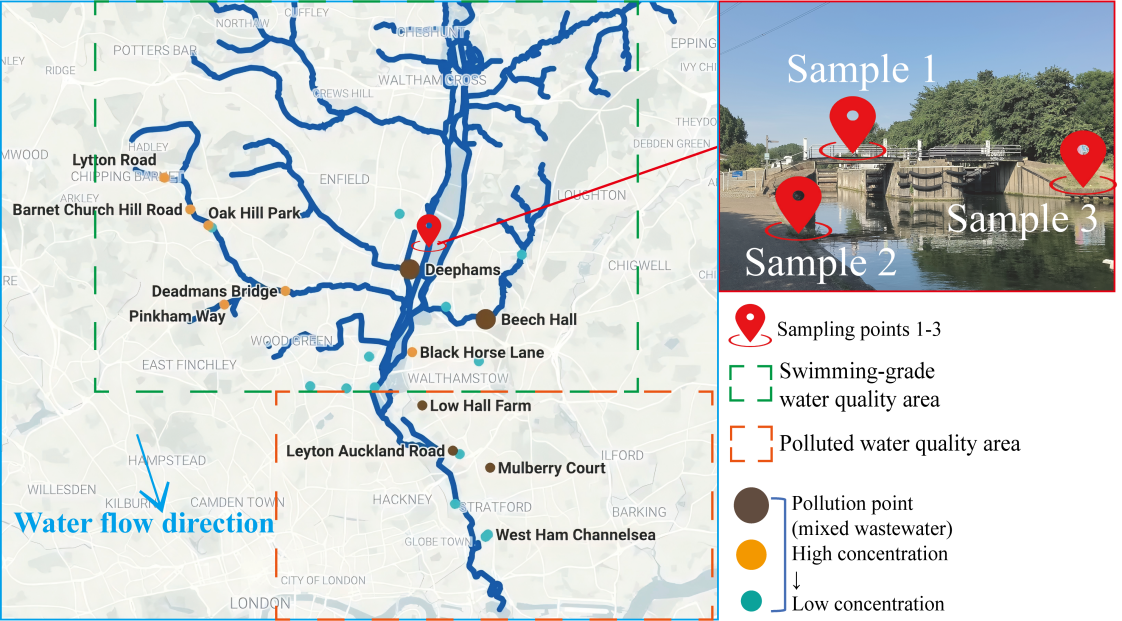


**Figure S14** Map of water quality and sampling locations in the Lea Valley. Data sources: (1) Pollution sites from Environment Agency, UK^[40]^. (2) Water quality zones from Environment Committee of London City Hall ^[41]^, while the drawing of these zones is only an estimate, as only a schematic diagram of the area is provided in the rare material instead of the specific boundaries.

Pollutants, particularly plastics, have long plagued most waterways in London, especially the Thames ^[42]^. However, during a personal communication with officials from the Lea Valley Regional Park Authority, it was confirmed that their robust, long-term water quality monitoring programme with UCL has consistently yielded negative results for plastic contamination or other pollution of high concentration. Environment Committee of London City Hall still defines the water quality of the section of the river where the sampling point was located as “swimming safe” in its latest report in 2025, as no harmful substances were detected, although the swimming is not permitted ^[41]^. In 2024, *Waltham Forest Echo* reported a long-term sewage leak downstream of the Lea Valley with the help from Thames Water (responsible for water treatment in London) and Environment Agency UK, but the upstream area where the sampling point was located was not affected ^[40]^. In this process, the pollution type is the mixed domestic sewage and there are no reports of plastic pollution until 2026.

**Photodegradation equipment and tensile tester**


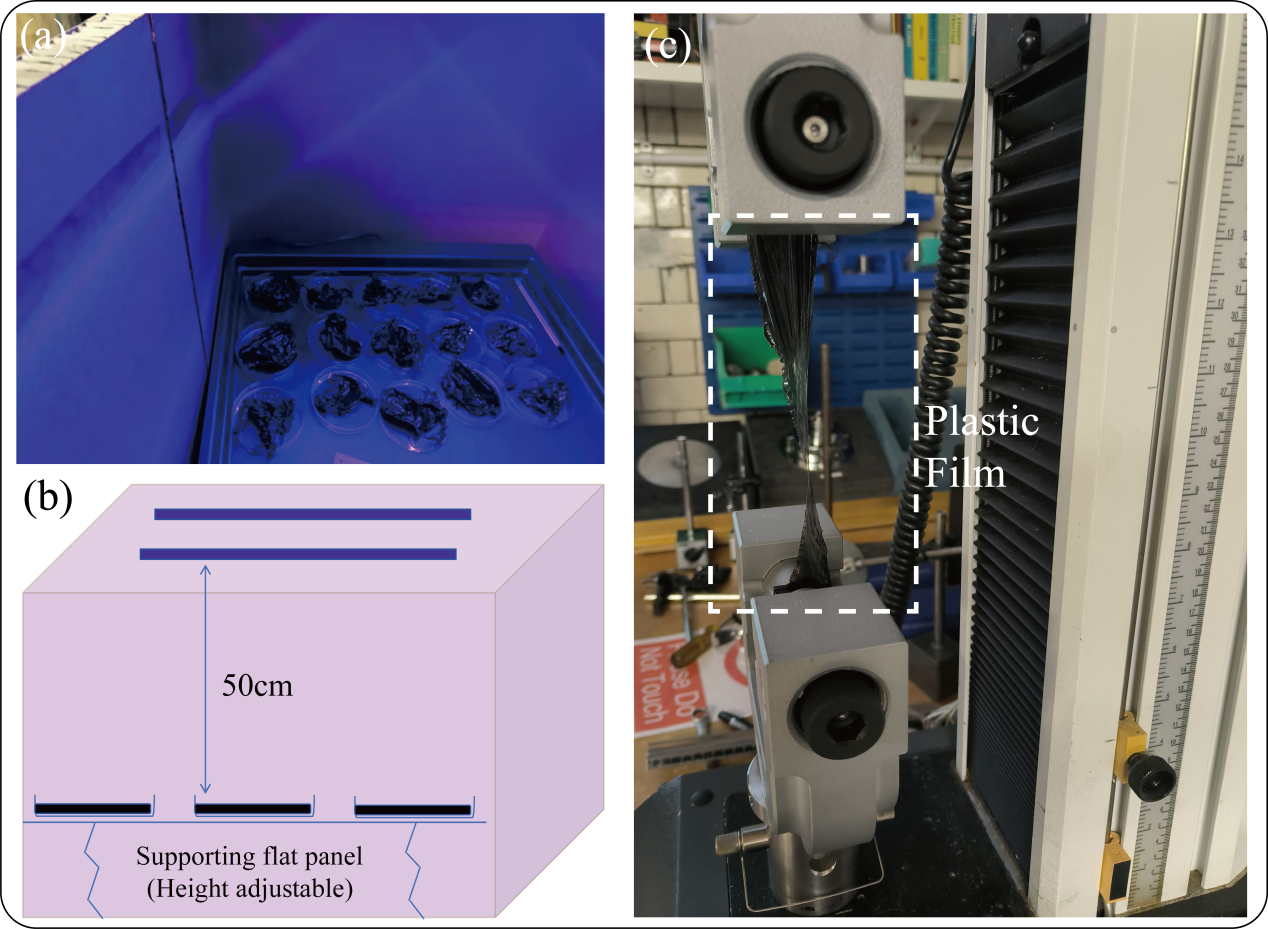


**Figure S15** Photodegradation experimental setup and schematic diagram, including (a) Photoreaction equipment; (b) Schematic diagram of photoreaction equipment; (c) Tensile strength test of plastic film.

Tensile tests were performed using a universal tensile testing machine in accordance with the international standard BS EN ISO 527-1:2019. This standard defines the method for determining the tensile properties of plastics. In this test, each film sample was securely clamped at both ends and stretched at a constant speed until it broke as shown in Figure S15(c). The machine continuously recorded the applied force and the corresponding elongation. From these data, key mechanical properties, such as tensile strength (the maximum stress the material can withstand while being stretched), were calculated to quantitatively evaluate how the degradation process affected the material’s strength and ductility.

**Section 4 Limitation and Outlook**

**Limitation**

Despite the promising findings of this proof‑of‑concept study, several limitations should be acknowledged.

1. Material characterisation. While PB‑NMs showed good dispersion and high surface area, we did not measure hydrodynamic size distribution, colloidal stability over time, or dissolution/leaching behaviour (including DOC release and mineral leaching). These parameters are essential for fully assessing the environmental mobility and catalytic availability of PB‑NMs under hydrated soil conditions. In addition, ash and mineral content were not quantified.
2. Mechanistic evidence. The proposed photocatalytic mechanism (e.g., radical generation, electron transfer) is supported only by indirect evidence from XPS and FT‑IR. Direct measurements (e.g., reactive oxygen species (ROS) scavenging assays, electron paramagnetic resonance (EPR) spectroscopy, band‑gap or photocurrent analysis, catalytic recycling tests) were not performed.
3. Functional gene inference. Microbial functional gene predictions were derived from 16S rRNA amplicon sequencing using PICRUSt2. These predictions represent potential functional capacity, not actual gene expression or enzyme activity. No metagenomics, metatranscriptomics, qPCR, or enzyme assays were conducted.
4. Generalisability. The results were obtained under controlled greenhouse conditions with a single plant species (alfalfa), a single NM synthesis batch, and a specific PE film. Performance under variable UV regimes, mixed plastic waste streams, different soil types, and real‑field conditions remain to be tested.

**Outlook**

Future work should prioritise field trials under natural UV radiation, diverse soil types, and mixed plastic waste conditions to validate scalability and ecological safety. On photodegradation mechanisms, direct evidence via ROS quenching, EPR spectroscopy, and photocurrent analysis is needed to confirm the proposed radical‑driven pathway, ideally using additive‑free or isotopically labelled plastics. For bioremediation, multi‑omics approaches (metagenomics, metatranscriptomics, or qPCR) should be applied to validate functional gene predictions, and plant growth phenotyping should adopt randomised sampling with larger per‑pot replication (n ≥ 5–6) and mixed‑effects models. Finally, comprehensive characterisation of PB‑NMs, including DLS, zeta potential, long‑term colloidal stability, leaching behaviour (DOC, mineral ions), and ash/mineral content, is essential to assess their environmental mobility and fate.

**References**

[1] ALTWALA A, MOKAYA R. Predictable and targeted activation of biomass to carbons with high surface area density and enhanced methane storage capacity [J]. Energy & Environmental Science, 2020, 13(9): 2967-78.

[2] MA X, CHEN R, ZHOU K, et al. Activated Porous Carbon with an Ultrahigh Surface Area Derived from Waste Biomass for Acetone Adsorption, CO2 Capture, and Light Hydrocarbon Separation [J]. ACS Sustainable Chemistry & Engineering, 2020, 8(31): 11721-8.

[3] LIU H, REN W, CIRIC L, BHATTI M. Phytotoxicological effects of phytosynthesized nanoparticles: A systematic review and meta-analysis [J]. Critical Reviews in Environmental Science and Technology, 2024: 1-21.

[4] JIANG Y, LIU Y, ZHANG Y, et al. Micro-Structure Determines the Intrinsic Property Difference of Bio-Based Nitrogen-Doped Porous Carbon-A Case Study [J]. Nanomaterials (Basel), 2020, 10(9).

[5] JIAO H, CUI M, YUAN S, et al. Carbon nanomaterials for co-removal of antibiotics and heavy metals from water systems: An overview [J]. J Hazard Mater, 2025, 489: 137566.

[6] SHEN J H, LI M M, CHU L F, et al. Effect mechanism of copper ions on photocatalytic activity of TiO2/graphene oxide composites for phenol-4-sulfonic acid photodegradation [J]. J Colloid Interface Sci, 2021, 586: 563-75.

[7] WANG J, WANG S. Preparation, modification and environmental application of biochar: A review [J]. Journal of Cleaner Production, 2019, 227: 1002-22.

[8] KUMAR A, KUMAR A, SHARMA G, et al. Biochar-templated g-C3N4/Bi2O2CO3/CoFe2O4 nano-assembly for visible and solar assisted photo-degradation of paraquat, nitrophenol reduction and CO2 conversion [J]. Chemical Engineering Journal, 2018, 339: 393-410.

[9] SCHWAB S T, BAUR M, NELSON T F, MECKING S. Synthesis and Deconstruction of Polyethylene-type Materials [J]. Chem Rev, 2024, 124(5): 2327-51.

[10] LI S, ZHANG S, XU J, et al. Photodegradation of polycyclic aromatic hydrocarbons on soil surface: Kinetics and quantitative structure-activity relationship (QSAR) model development [J]. Environ Pollut, 2024, 345: 123541.

[11] YAN X, AN J, ZHANG Y, et al. Photochemical degradation in natural attenuation of characteristics of petroleum hydrocarbons (C(10)-C(40)) in crude oil polluted soil by simulated long term solar irradiation [J]. J Hazard Mater, 2023, 460: 132259.

[12] LOZANO Y M, GORDILLO‐ROCHA H, WALDMAN W R, RILLIG M C. Photodegradation modifies microplastic effects on soil properties and plant performance [J]. Journal of Applied Ecology, 2023, 61(1): 13-24.

[13] CHAE E, CHOI S S. Influence of Molecular Weight and Temperature on the Pyrolysis Behavior of Polyethylene [J]. Polymers (Basel), 2025, 17(5).

[14] PARK K-B, KIM J-S. Pyrolysis products from various types of plastics using TG-FTIR at different reaction temperatures [J]. Journal of Analytical and Applied Pyrolysis, 2023, 171.

[15] LU J, VEKSHA A, LISAK G. Conversion of municipal sewage sludge into biogenic multi-walled carbon nanotubes and hydrogen using X-Mo/MgO (X = Co, Fe, Ni) catalysts through pyrolysis-chemical vapor deposition process [J]. Chemical Engineering Journal, 2024, 496.

[16] TAN K Q, LIM X X, MARIMUTHU A, et al. Upcycling polymeric wastes into multi-walled carbon nanotubes via dual-stage pyrolysis: advancing sustainable resource recovery [J]. Waste Manag, 2025, 206: 115071.

[17] BU Y, GUO F, LI K, et al. High-temperature pyrolysis behavior and structural evolution mechanism of graphene oxide: A ReaxFF molecular dynamics simulation [J]. Applied Surface Science, 2022, 593.

[18] WANG Y, WANG S, GUO F, et al. Thermal decomposition behavior of GO/TKX-50 with different oxidation degree [J]. Journal of Thermal Analysis and Calorimetry, 2023, 148(24): 13825-36.

[19] MAKOWSKA M, DZIOSA K. Influence of different pyrolysis temperatures on chemical composition and graphite-like structure of biochar produced from biomass of green microalgae Chlorella sp [J]. Environmental Technology & Innovation, 2024, 35.

[20] AL-RABAIAI A, MENEZES-BLACKBURN D, AL-ISMAILY S, et al. Customized biochar for soil applications in arid land: Effect of feedstock type and pyrolysis temperature on soil microbial enumeration and respiration [J]. Journal of Analytical and Applied Pyrolysis, 2022, 168.

[21] OKTAVIANA A A, HERMANA J, SYAFEI A D, HSI H C. Effect of pyrolysis temperature of domestic sewage sludge biochar on CO2 adsorption [J]. Results in Engineering, 2025, 26.

[22] BRANDL F, BERTRAND N, LIMA E M, LANGER R. Nanoparticles with photoinduced precipitation for the extraction of pollutants from water and soil [J]. Nat Commun, 2015, 6: 7765.

[23] HOU W C, BEIGZADEHMILANI S, JAFVERT C T, ZEPP R G. Photoreactivity of unfunctionalized single-wall carbon nanotubes involving hydroxyl radical: chiral dependency and surface coating effect [J]. Environ Sci Technol, 2014, 48(7): 3875-82.

[24] SHI X, WANG Z, LIU S, et al. Scalable production of carboxylated cellulose nanofibres using a green and recyclable solvent [J]. Nature Sustainability, 2024, 7(3): 315-25.

[25] MONDAL N K, KUNDU S, DEBNATH P, et al. Effects of polyethylene terephthalate microplastic on germination, biochemistry and phytotoxicity of Cicer arietinum L. and cytotoxicity study on Allium cepa L [J]. Environ Toxicol Pharmacol, 2022, 94: 103908.

[26] LUO Y, LI L, FENG Y, et al. Quantitative tracing of uptake and transport of submicrometre plastics in crop plants using lanthanide chelates as a dual-functional tracer [J]. Nat Nanotechnol, 2022, 17(4): 424-31.

[27] LIU H, CIRIC L, BHATTI M. Phytoremediation of organic pollution using leguminous plants and auxiliary additives: principles and advantages [J]. Chemical and Biological Technologies in Agriculture, 2025, 12(1).

[28] MA S, HUA Z, TANG C, et al. Root Meristem Maintenance Mechanisms are Key to Plant Defense Against Nanoplastics [J]. Adv Sci (Weinh), 2025: e11837.

[29] BILAL M, ADEEL M, RASHEED T, et al. Emerging contaminants of high concern and their enzyme-assisted biodegradation - A review [J]. Environ Int, 2019, 124: 336-53.

[30] DAI Y, LIU R, CHEN J, LI N. Bioremediation of HMW-PAHs-contaminated soils by rhizosphere microbial community of Fire Phoenix plants [J]. Chemical Engineering Journal, 2022, 432.

[31] ZHANG X, CHI G, XIA H, et al. Deciphering the Helianthus annus rhizosphere soil biodiversity under petroleum hydrocarbon compounds contamination [J]. Process Safety and Environmental Protection, 2023, 178: 795-806.

[32] XU Y, CHAN F K S, JOHNSON M, et al. Microplastic pollution in Chinese urban rivers: The influence of urban factors [J]. Resources, Conservation and Recycling, 2021, 173.

[33] WANG K, FLURY M, KUZYAKOV Y, et al. Aluminum and microplastic release from reflective agricultural films disrupt microbial communities and functions in soil [J]. J Hazard Mater, 2025, 491: 137891.

[34] ALLAIN A, ALEXIS M A, BRIDOUX M C, et al. The specific molecular signature of dissolved organic matter extracted from different arctic plant species persists after biodegradation [J]. Soil Biology and Biochemistry, 2024, 193.

[35] FENG L J, SUN X D, ZHU F P, et al. Nanoplastics Promote Microcystin Synthesis and Release from Cyanobacterial Microcystis aeruginosa [J]. Environ Sci Technol, 2020, 54(6): 3386-94.

[36] REN W, LIU H, MAO T, et al. Enhanced remediation of PAHs-contaminated site soil by bioaugmentation with graphene oxide immobilized bacterial pellets [J]. J Hazard Mater, 2022, 433: 128793.

[37] HUANG D, XU Y, LEI F, et al. Degradation of polyethylene plastic in soil and effects on microbial community composition [J]. J Hazard Mater, 2021, 416: 126173.

[38] TANIGUCHI I, YOSHIDA S, HIRAGA K, et al. Biodegradation of PET: Current Status and Application Aspects [J]. ACS Catalysis, 2019, 9(5): 4089-105.

[39] FU F, LONG B, HUANG Q, et al. Integrated effects of residual plastic films on soil-rhizosphere microbe-plant ecosystem [J]. J Hazard Mater, 2023, 445: 130420.

[40] DURRANT W, CRACKNELL J. Hundreds of sewage spills into River Lea and its tributaries last year [N]. Waltham Forest Echo, 2024-.

[41] POLANSKI Z. Swimmable rivers: Towards clean and healthy waterways in London [Z]//COMMITTEE E. London City Hall; Environment Committee. 2025

[42] ROWLEY K H, CUCKNELL A C, SMITH B D, et al. London's river of plastic: High levels of microplastics in the Thames water column [J]. Sci Total Environ, 2020, 740: 140018.
